# Parallels between the Developing Vascular and Neural Systems: Signaling Pathways and Future Perspectives for Regenerative Medicine

**DOI:** 10.1002/advs.202101837

**Published:** 2021-10-24

**Authors:** Idoia Elorza Ridaura, Stefano Sorrentino, Lorenzo Moroni

**Affiliations:** ^1^ Complex Tissue Regeneration Department MERLN Institute for Technology‐Inspired Regenerative Medicine Maastricht University Universiteitssingel 40 Maastricht 6229ER The Netherlands; ^2^ CNR Nanotec – Institute of Nanotechnology Campus Ecotekne, via Monteroni Lecce 73100 Italy

**Keywords:** growth cone, neural stem cells, neurovascular unit, stem cell therapy, tip cells

## Abstract

Neurovascular disorders, which involve the vascular and nervous systems, are common. Research on such disorders usually focuses on either vascular or nervous components, without looking at how they interact. Adopting a neurovascular perspective is essential to improve current treatments. Therefore, comparing molecular processes known to be involved in both systems separately can provide insight into promising areas of future research. Since development and regeneration share many mechanisms, comparing signaling molecules involved in both the developing vascular and nervous systems and shedding light to those that they have in common can reveal processes, which have not yet been studied from a regenerative perspective, yet hold great potential. Hence, this review discusses and compares processes involved in the development of the vascular and nervous systems, in order to provide an overview of the molecular mechanisms, which are most promising with regards to treatment for neurovascular disorders. Vascular endothelial growth factor, semaphorins, and ephrins are found to hold the most potential, while fibroblast growth factor, bone morphogenic protein, slits, and sonic hedgehog are shown to participate in both the developing vascular and nervous systems, yet have not been studied at the neurovascular level, therefore being of special interest for future research.

## Graphical abstract

Blood vessels and neurons grow along one another and can influence each other's maturation, both during embryonic development and regeneration in adulthood. Understanding the molecular processes behind these events could improve current treatments for neurovascular disorders. Consequently, this review investigates the signaling pathways and their promises for regenerative medicine.

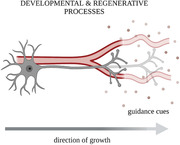

## 1 Introduction

**Figure 1:**
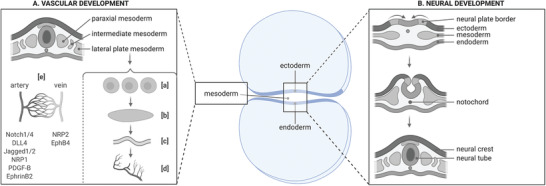
Formation of vascular and nervous systems during embryonic development. A) Blood vessels arise from the lateral plate mesoderm. a) Angioblast precursors aggregate, forming b) blood islands. c) Vasculogenesis forms endothelial tubes. d) Mature blood vessels then develop through angiogenesis. e) Finally, blood vessels differentiate into arteries and veins, expressing different markers. Markers such as NRP1 and NRP2 can be used to distinguish arteries from veins. EphrinB2 is also expressed on arteries, while EphB4 can be found on veins. B) The nervous system is derived from the ectoderm. During neurulation, the notochord triggers the ectoderm to fold. The resulting neural tube and crest later develop into to the central and peripheral nervous systems, respectively.

The vascular and nervous systems, respectively taking care of blood transport and coordinating sensory and motor information, are essential to the human body and share many similarities. The first begins developing very early after gastrulation. The heart arises from an area of the mesoderm known as the first heart field, forming the heart tube during the third week of development.^[^ ^1^ ^]^ Blood vessels develop from blood islands in the yolk sac and angioblast precursors in the head mesenchyme,^[^ ^1^ ^]^ which aggregate and elongate to form tube‐like structures.^[^ ^1^ ^]^ While the heart starts developing, the ectoderm undergoes neurulation.^[^ ^2^ ^]^ Throughout the third and fourth weeks, it folds and forms the neural tube from which the central nervous system (CNS) will derive (**Figure** **1**).^[^ ^2^ ^]^ After folding of the neural tube, a pool of cells originally located in the neural plate border delaminate and migrate in a rostral to caudal direction as neural crest cells from which the peripheral nervous system (PNS) will arise.^[^ ^3^ ^]^

At the anatomical level, arteries and nerves often run along one another, making their organization highly similar.^[^ ^4^, ^5^ ^]^ At the cellular level, tip cells and growth cones are comparable both in their morphological structures and functions.^[^ ^6^ ^]^ Tip cells are specialized endothelial cells guiding developing blood vessels, while growth cones are located at the leading ends of growing axons. At the molecular level, tip cells and growth cones have been shown to respond to similar guidance cues.^[^ ^5^, ^6^ ^]^ Semaphorins, slits, ephrins, and netrins were discovered, in the 1990s.^[^ ^5^ ^]^ Nowadays, the list of signaling molecules involved in both systems continues to grow. Furthermore, some signaling molecules studied in the developing neural and vascular systems have also been investigated, in the context of neurovascular development (**Table** **1**).

**Table 1:** Overview of signaling molecules. a) List of molecules discussed in this review. b) Systems within which each molecule is involved—vascular, neural, or neurovascular. c,d) Main roles in the development and regeneration of said systems. e) Main findings on their application in the context of regenerative medicine

a) Molecules	b) Systems involved	c) Roles in development	d) Roles in regeneration	e) Regenerative medicine
VEGF/PIGF/HIF1*α*	Vascular, neural, neurovascular	VEGF is involved in production of haematopoietic precursors and angioblasts, as well as the formation of blood vessels.^[^ ^22^ ^]^ Its levels increase in response to hypoxia, which is mediated by HIF1*α*.^[^ ^1^ ^]^ Receptors VEGFR2 and VEGFR1 are selectively expressed in tip and stalk cells,^[^ ^25^ ^]^ and contribute to their differentiation.^[^ ^27^ ^]^ VEGF can also attract tip cells.^[^ ^27^ ^]^ In the CNS, VEGF promotes axonal and dendrite growth.^[^ ^52^, ^53^, ^54^, ^55^, ^56^ ^]^ In the PNS, it attracts motor neurons to the dorsal root ganglia, and modulates their velocity and size.^[^ ^58^, ^59^ ^]^ It also promotes Schwann cell proliferation and migration.^[^ ^60^ ^]^ HIF1*α* is involved in survival and proliferation of PNS neurons.^[^ ^61^ ^]^ VEGF is also involved in the interaction between both developing systems. Neural cells expressing the molecule could guide tip cells and affect vascular differentiation.^[^ ^6^, ^105^ ^]^ Endothelial cell‐derived VEGF promoted proliferation, differentiation, and migration of neurons and their precursors.^[^ ^6^ ^]^	A specific VEGF isoform, VEGF165, induced endothelial differentiation of adult multipotent cells—hMAPCs.^[^ ^27^ ^]^ VEGF is involved in peripheral nerve regeneration.^[^ ^95^ ^]^ Immunohistochemical evidence suggested an autocrine pathway located on Schwann cells.^[^ ^95^ ^]^ It also promotes NSC differentiation into neuroblasts.^[^ ^134^ ^]^ VEGF appears to promote blood‐brain barrier maintenance,^[^ ^133^ ^]^ but has also been found to reduce its integrity.^[^ ^136^ ^]^ Neural‐derived VEGF also induced arterial differentiation.^[^ ^135^ ^]^	In humans after ALS, intracerebroventricular administration of VEGF in ALS patients results in detectable CSF levels.^[^ ^156^ ^]^ In humans suffering from stroke, NBP promotes recovery from acute cerebral infarction, increasing VEGF levels in serum.^[^ ^157^ ^]^ In mice after stroke, worse stroke‐related brain damage correlated with increased levels of VEGFA, which were both reversed by Uric Acid.^[^ ^159^ ^]^ In mice after PAD, VEGF‐expressing MSC administration resulted in a significant increase in CBF restoration.^[^ ^182^ ^]^ VEGF165‐binding heparan sulfate sugars enhanced recovery from ischaemia in rats.^[^ ^158^ ^]^
Delta/Notch	Vascular, neurovascular	Several molecules of the Notch pathway are selectively expressed on arterial and venous walls and thus probably play a role in blood vessel differentiation.^[^ ^1^, ^27^ ^]^ Endothelial cell‐derived Notch maintains NSCs in their quiescent state.^[^ ^117^ ^]^	Notch mediates VEGF's effects on endothelial differentiation of multipotent cells, by defining whether they will turn into arterial or venous cells.^[^ ^27^ ^]^ Notch signaling maintains endothelial cell quiescence in adulthood.^[^ ^26^ ^]^ Notch3 also promoted pericyte maturation,^[^ ^137^ ^]^ and is involved in pericyte‐dependent blood‐brain barrier regeneration.^[^ ^136^ ^]^	
FGF/FGFBP	Vascular, neural	FGFs are believed to support endothelial cell proliferation, migration, and differentiation.^[^ ^30^ ^]^ However, contradicting results have been found regarding their important in angiogenesis.^[^ ^30^, ^31^ ^]^ FGFBPs are believed to be important in the wiring of the brain and development of cortical functions. However, their specific roles and mechanisms are unclear.^[^ ^62^ ^]^ In the PNS, FGFBP1 is involved in neuromuscular junction formation, while FGF7, FGF10, and FGF22 enhance maturation of presynaptic regions.^[^ ^62^ ^]^	FGF is involved in repair‐associated angiogenesis, contributing to vessel formation in models of ischaemia.^[^ ^30^ ^]^	Intra‐arterial administration of FGF2 protein, FGF1‐encoding adenoviruses, and FGF2 bioreactors increased blood vessel formation.^[^ ^30^ ^]^ In mice, FGF2 overexpression resulted in Schwann cell proliferation and enhanced myelination, and doubled the number of regenerating axons.^[^ ^32^ ^]^ FGF administration improved reocvery after spinal cord injury in rats.^[^ ^208^ ^]^
ANGPT/TIE	Vascular	ANGPT2 and TIE2 are respectively expressed in tip and stalk cells and are involved in endothelial cell migration.^[^ ^26^ ^]^ Inhibition of TIE2 leads to unstable vessels, which might promote remodeling during regeneration.^[^ ^1^ ^]^		
SEMA/Plexins/NRP	Vascular, neural, neurovascular	SEMA3A, SEMA3B, SEMA3D, SEMA3E, SEMA3F seem to have anti‐angiogenic effects, while SEMA4D, SEMA5A, and SEMA6A promote angiogenesis.^[^ ^25^ ^]^ NRPs enhance vascularisation.^[^ ^1^ ^]^ NRP1 and NRP2 are respectively expressed on arteries and veins.^[^ ^27^ ^]^ SEMAs are involved in synapse formation, neuronal apoptosis, dendrite growth, and axonal guidance.^[^ ^34^, ^64^ ^]^ SEMA3E attracts growth cones, while SEMA3A causes surround repulsion.^[^ ^32^, ^33^, ^34^ ^]^ GnRH hormones have shown special affinity for SEMA3A guidance.^[^ ^34^ ^]^ SEMA3E produced by neural cells might signal to endothelial cells and guide their development.^[^ ^119^, ^120^ ^]^	SEMAs are believed to enhance cell survival and promote growth factor secretion, during neural regeneration.^[^ ^93^ ^]^ SEMA5A was found to participate in both vascular and nervous regeneration, after sciatic nerve transection.^[^ ^111^ ^]^	
PDGF‐B	Vascular	PDGF‐B is expressed in higher levels in tip than stalk cells.^[^ ^36^ ^]^ It is also involved in pericyte recruitment and retention.^[^ ^37^ ^]^		PDGF‐B injections improved recovery after damage to the nervous system.^[^ ^162^ ^]^
SMAD6	Vascular	SMAD6 responds to haemodynamic factors, is mainly expressed in big arteries, and plays a role in endothelial junction stabilization.^[^ ^38^ ^]^		
TGF‐*β*	Vascular, neurovascular	The TGF‐*β* family regulates a vast array of molecules involved in angiogenesis – including VEGF, FGF, and ANGPT.^[^ ^41^ ^]^ It is also involved in tip cell selection,^[^ ^35^ ^]^ blood vessel differentiation, production of extracellular matrix, and recruitment of supporting cells.^[^ ^1^ ^]^ Overall, it could modulate a balance between endothelial proliferation and vessel stabilization. In the CNS, TGF‐*β* is activated by neural progenitors and involved in cerebral vascularization.^[^ ^6^ ^]^	During axonal recovery, TGF‐*β* is released by macrophages and attracts Schwann cells to the nerve stump. TGF‐*β* enhances their mitotic activity,^[^ ^93^ ^]^ and they in turn guide the axon's regrowth.^[^ ^32^ ^]^ Pericyte‐derived TGF‐*β* promotes blood‐brain barrier integrity by supporting tight junctions between endothelial cells, increasing extracellular protein secretion, and enhancing N‐cadherin expression between pericytes and endothelial cells.^[^ ^136^ ^]^	In humans after stroke, administration of PA with GS induced an increase in TGF‐*β*1 levels that correlated with reduced incidence of symptomatic intracerebral haemorrhage.^[^ ^209^ ^]^
BMP	Vascular, neural	BMPs are involved in tip cell differentiation.^[^ ^26^ ^]^ BMP6 and BMP7 promote vessel sprouting, while BMP9 and BMP10 enhance vessel quiescence and stability.^[^ ^38^ ^]^ BMP inhibition is essential for the formation of the neural plate.^[^ ^2^ ^]^ Later, BMP6 and BMP7 control the differentiation of specific interneurons in the spinal cord.^[^ ^32^ ^]^ BMP7 regulates growth cone motility, but whether it attracts or repels growth cones is still unclear.^[^ ^32^ ^]^	Following spinal cord injury, BMP was shown to be negatively involved in regeneration in mice. Decreased levels through genetic mutations and BMP antagonists result in improved functional recovery.^[^ ^32^ ^]^ Presumably, BMP2 and BMP4 increase the presence of oligodendrocytes and astrocytes, which hinder recovering axons.^[^ ^32^ ^]^ However, BMP2 and BMP7 resulted in improved regeneration of facial nerves and spinal cord, in rats.^[^ ^68^ ^]^ BMP is involved in pericyte‐dependent blood‐brain barrier regeneration.^[^ ^136^ ^]^	
Cadherins	Vascular	VE‐cadherins are involved in endothelial cell adherens junctions.^[^ ^23^ ^]^ Their internalization in tip cells is essential to promote motility.^[^ ^38^ ^]^		
Slit/Robo	Vascular, neural	Robo4 seems to be involved in cell migration, by stimulating filipodia formation in tip cells, and vessel stabilization through blockade of VEGF signaling.^[^ ^25^ ^]^ Slit acts as a repulsive cue in neuronal development.^[^ ^33^ ^]^ Slit and Robo also control midline crossing, through isoforms Robo3.1 and Robo3.2.^[^ ^33^ ^]^		
RA	Vascular, neurovascular	RA is involved in early‐stages vasculogenesis and recruitment of smooth muscle cells to large vessels.^[^ ^31^ ^]^ RA is involved in neural control of cerebral vascular development, especially in the formation of the blood‐brain barrier.^[^ ^121^ ^]^ It interacts with VEGF and Wnt.^[^ ^31^, ^122^ ^]^		
COUP‐TFII	Vascular	COUP‐TFII promotes blood vessel differentiation by enhancing the expression of venous markers.^[^ ^27^ ^]^		
Shh	Vascular, neural	Contradicting results have not clarified whether Shh promotes angiogenesis or vessel quiescence.^[^ ^42^ ^]^ Overall, it is believed to play a role in vessel branching, arterial differentiation, and pericyte recruitment.^[^ ^42^ ^]^ Shh regulates the development of the spinal cord,^[^ ^2^ ^]^ acts as a chemorepulsive cue, and guides commissural axons via a cross‐talk with Wnt signaling.^[^ ^32^ ^]^		Administration of Hh ligands increased capillary density and promoted muscle perfusion in aged mice.^[^ ^42^ ^]^ Whether Shh‐induced angiogenesis involves Hh signaling is still unclear.^[^ ^42^ ^]^ Exogenous Shh reduced neural damage related to traumatic brain injury in rats.^[^ ^175^ ^]^
Eph/Ephrins	Vascular, neural, neurovascular	EphrinB2 and EphB4 are respectively expressed in arterial and venous endothelial cells.^[^ ^1^ ^]^ Ephrins also mediate the effects that haemodynamic factors have on lumen diameter.^[^ ^27^ ^]^ Ephrins modulate topographic mapping of the tectum, act as short‐range guidance cues, guide commissural axons, and regulate dendritic morphology and synapse formation.^[^ ^32^, ^33^ ^]^ Ephrins are involved in blood‐brain barrier development and mediate cross‐talk between glial cells and developing vasculature in the CNS.^[^ ^123^ ^]^	EphrinB2 levels are naturally upregulated in arterial vasculature following ischaemia, and promote angiogenesis.^[^ ^27^ ^]^ In neural regeneration, Ephrins are believed to enhance cell survival and promote growth factor secretion.^[^ ^93^ ^]^ EphB2 and EphB3 were found to participate in both vascular and nervous regeneration, after sciatic nerve transection.^[^ ^111^ ^]^	EphrinB2 administration resulted in enhanced angiogenesis.^[^ ^27^ ^]^
Wnt	Neural, neurovascular	Wnt regulates growth cone guidance, promotes PNS specification, and controls neural differentiation.^[^ ^2^, ^66^ ^]^ It is also involved in cellular migration, proliferation, and myelination,^[^ ^68^ ^]^ and attracts midline‐crossing axons.^[^ ^32^ ^]^ Wnt is expressed by neurons and their precursors, and signals to endothelial cells in the CNS.^[^ ^31^ ^]^ It is also involved in blood vessel and blood‐brain barrier formation.^[^ ^6^ ^]^	Wnt and its canonical pathway are activated after spinal cord and optic nerve injuries, and are believed to promote functional recovery of injured axons.^[^ ^32^ ^]^	Wnt administration resulted in improved recovery after ischaemic and haemorrhagic stroke.^[^ ^168^ ^]^
Netrin	Neural	Netrins are involved in midline‐crossing through DCC.^[^ ^70^, ^71^, ^72^ ^]^ Netrin‐1 also acts as a guidance cue.^[^ ^32^ ^]^ In the PNS, Netrins seem of special importance in motor neuron guidance.^[^ ^74^ ^]^	Netrins are believed to enhance cell survival and promote growth factor secretion, during neural regeneration.^[^ ^93^ ^]^	
NGF	Neural	NGF sustains innervated neurons during selective synapse elimination,^[^ ^5^ ^]^ and modulates local chemoattraction.^[^ ^75^ ^]^	In neural regeneration, activated macrophages at the injury site induce the secretion of NGF by Schwann cells.^[^ ^93^ ^]^ NGF has been shown to be expressed by pericytes,^[^ ^136^, ^139^ ^]^ and support neurite outgrowth.^[^ ^68^ ^]^	NGF improves axonal regeneration and locomotor function.^[^ ^32^ ^]^
Reelin	Neural	Reelin controls cellular migration and positioning in the developing cortex, by acting as a gradient and guiding radially migrating neurons.^[^ ^77^ ^]^		
JNK	Neural	JNKs are involved in neuronal differentiation, guidance of commissural axons, and dendrite and axon formation.^[^ ^78^ ^]^ JNK1 also induces midline crossing in commissural axons.^[^ ^78^ ^]^	Although JNK triggers neuronal degeneration in disorders such as ischaemia, epilepsy, or Alzheimer's disease,^[^ ^78^ ^]^ it seems to be necessary in nerve regeneration, especially for neurite elongation and neuritogenesis.^[^ ^78^, ^96^ ^]^	
CAMs	Neural	NCAMs allow axons to adhere to each other in order to develop together.^[^ ^32^ ^]^		In humans after stroke, enlimomab, an ICAM‐1 antibody, induces worse recovery, fewer symptom‐free patients, more infections, and fever.^[^ ^169^ ^]^
NTs	Neural	NT‐3 acts as a chemoattractant in the CNS.^[^ ^32^, ^68^ ^]^ In the PNS, it is important for the development of sympathetic neurons.^[^ ^79^, ^80^, ^81^ ^]^ In the enteric nervous system, it promotes precursor cell survival and differentiation.^[^ ^82^ ^]^	NT‐3 attracted recovering adult rat corticospinal fibers and induced regeneration.^[^ ^32^ ^]^ NT‐4 and NT‐5 have also been shown to have positive effects on recovering nerves, preventing cell death, promoting functional reinnervation, and increasing axonal diameter, number, and myelin thickness^[^ ^68^ ^]^ Following a stroke, pericytes were shown to have increased NT‐3 expression.^[^ ^136^ ^]^	
BDNF	Neural, neurovascular	BDNF promotes neuronal survival and differentiation, as well as synapse formation and axon guidance.^[^ ^32^, ^68^ ^]^ Its activity has been related to increased protein synthesis in neurons, and filopodia number and length in growth cones.^[^ ^32^, ^85^ ^]^ BDNF is expressed by endothelial cells in the CNS and acts as a migration cue and neuroprotective signal.^[^ ^102^, ^125^, ^126^ ^]^	Some studies found BDNF to improve recovery following neural damage,^[^ ^68^, ^93^ ^]^ while others observed no improvements.^[^ ^32^ ^]^ The difference in injury location might explain the different outcomes. Following a stroke, pericytes were shown to have increased BDNF expression.^[^ ^136^ ^]^	In humans after stroke, Cytoflavin showed increased levels of BDNF, and improved cognitive functions after recovery.^[^ ^170^ ^]^ Semax and aerobic exercise were shown to increase BDNF levels in patients recovering from stroke.^[^ ^171^ ^]^
GDNF	Neural	GDNF acts as a chemoattractant,^[^ ^68^ ^]^ and enhances neurite arborization and extension.^[^ ^86^ ^]^	GDNF has been shown to be produced following neuronal injuries, and to promote recovery.^[^ ^93^, ^136^, ^139^ ^]^ It prevented retrograde motor neuron loss and atrophy.^[^ ^68^ ^]^	Decreased GDNF levels were observed in patients suffering from chronic denervation, indicating a possibility for treatments involving GDNF.^[^ ^32^ ^]^ Treatment with GDNF prevented SEMA3A‐mediated surround repulsion.^[^ ^87^ ^]^
GABA	Neurovascular	GABA modulates cell proliferation, neuroblast migration, and dendritic maturation.^[^ ^128^ ^]^ In the brain, it is involved in the formation of a functional cortical structure: endothelial cells across different areas express different receptors, which guide neurons to their destination.^[^ ^6^ ^]^ It is also believed to enhance CNS angiogenesis, especially promoting vascular density.^[^ ^129^ ^]^		In animal models of stroke, GABA receptor agonists were shown to decrease infarct size, and improve functional outcome. Usage limited by sedative side‐effects.^[^ ^173^ ^]^
RAS	Neurovascular	Glial cells produce AngII, the main ligand of RAS, while its receptors can be found on neurons, glia cells, and blood vessels.^[^ ^100^ ^]^ The system seems to be involved in regulation of glial function, microglial activation, modulation of neuronal function, and blood vessel constriction in the retina.^[^ ^100^ ^]^	AngI and receptor TIE2 are involved in endothelial regeneration. They enhance cell survival and migration, cellular adhesions, pericyte coverage, and tubulogenesis—the formation of tubes in vasculogenesis.^[^ ^136^, ^137^ ^]^ Pericyte‐derived AngI also increases blood‐brain barrier integrity.^[^ ^136^ ^]^	In rats after stroke, Candesartan (ARB), an AT2‐R agonist, decreases cognitive impairment 7 days post‐stroke.^[^ ^174^ ^]^

A variety of disorders whose pathophysiology combines the vascular and neural systems – including cardiovascular and neurodegenerative diseases (NDs) – are leading causes of long‐term disability.^[^ ^7^ ^]^ NDs are defined by progressive and irreversible neuronal cell death leading to cognitive or motor impairments according to the brain area affected. Despite the differences, NDs share some distinct features such as chronic and progressive nature, time prevalence dependence, selective vulnerability, and misfolded protein aggregation. This last one is disease‐specific but is able to trigger a general neuroinflammatory reaction within the brain through glial cells activation. If the inflammatory response so created cannot find a resolution it becomes detrimental, altering neuronal tissue functioning and the integrity of the blood brain barrier (BBB). Novel emerging data have demonstrated how vascular dysfunctions can appear as early events in the pathogenesis of several NDs, including Alzheimer, Parkinson and amyotrophic lateral sclerosis (ALS),^[^ ^8^ ^]^ re‐centering the attention of the neurovascular axis in the pathogenesis of these illnesses. In this regard, the BBB is involved in the clearance of neurotoxic proteins and maintenance of the balance between their production and removal from CNS.^[^ ^9^ ^]^ Vascular dysfunctions and BBB leakages could therefore lead to the accumulation of toxic species within the brain and allow the entrance of immune cells which exacerbate the inflammatory worsening of the disease. Understand the relationship between neuronal and vascular systems is therefore essential to disclose the molecular mechanism linked to NDs pathogenesis.

Cardio‐ and cerebrovascular pathologies are leading causes of death and disability worldwide. To discuss the pathological relation between vascular and neuronal components, stroke for CNS and peripheral artery disease (PAD) for PNS will be proposed as explicative examples.

PAD is an abnormal narrowing of peripheral arteries referred to as an atherosclerosis event that impairs blood flow in vessels outside the heart, most commonly affecting arteries of the lower limbs. Despite its high prevalence, extremely marked in Southeast Asia and Western Pacific regions, patients affected by PAD often remain underdiagnosed because of the frequent asymptomatic onset of the disease.^[^ ^10^ ^]^ Clinically, there are two classic manifestations of PAD: intermittent claudication and critical limb ischemia (CLI). Individuals with intermittent claudication typically present leg pain, aching, cramping, or fatigue during ambulation that are relieved by rest. Contrarily, those with CLI suffer from chronic ischemic rest pain often associated with ulceration or gangrene. This last form of PAD is associated with a high risk of limb amputation, cardiovascular events, and death.^[^ ^11^ ^]^ Although controversial, this chronic PAD is believed to cause axonal degeneration and result in axonal polyneuropathy.^[^ ^12^, ^13^ ^]^ Nerve damage has also been suggested to reinforce the gravity of arterial occlusion,^[^ ^14^ ^]^ suggesting a possible bidirectional causality. However, despite both processes being related, the mechanism connecting them is still unclear. Moreover, unlike stroke, the molecular mechanisms driving the pathology remain largely unknown, and aside from the classic cardiovascular risk factors such as diabetes, hypertension, smoking, and aging, no other seems to contribute to PAD development. Currently, there are no effective medical treatments addressing the key issues in PAD, and patients are treated for cardiovascular risk factors as well as poor functional capacity.^[^ ^15^, ^16^ ^]^

Strokes are mainly divided into ischemic and haemorrhagic, with the firsts caused by arterial occlusions while the seconds by the leaking of blood vessels. Ischaemic strokes are the most represented, around 70% of all strokes globally, and are mostly caused by a thromboembolic event, such as artery atherosclerosis or cardiac diseases.^[^ ^17^ ^]^ The reduction or the lack of cerebral blood flow (CBF) followed by the thromboembolic event have dramatic and rapid consequences that affect neuronal and glial function in addition to vascular alterations and inflammation. Due to its poor ability to store energy, the nervous system is particularly vulnerable to a lack of ATP and relies on a continoues apport of oxygen and metabolites from CBF. When this supply is interrupted, as in stroke, the neuronal function is impaired leading to a sudden and massive depolarization with the release of neurotransmitters. One of them, glutamate, is particularly dangerous if not readily removed, and when accumulated in the extracellular space induces excitotoxicity leading to neuronal cell death, release of oxidative species, BBB disruption, and inflammation. A remarkable point when discussing stroke is its recovery. The recovery of stroke depends on several factors such as the time of intervention, the extension of the area affected, and the type of therapy. Biologically, recovery is based on neuroplasticity, which is the ability to readapt the neuronal network to reinnervate the area affected, through neurogenesis and angiogenesis. Post‐ischemic angiogenesis contributes to neuronal remodeling at both physical and chemical level. Blood vessels act as a scaffold for neural stem cells and guide their migration through gradients of oxygen and nutrients toward the regeneration site, as well as serving as a guide for sprouting axons. Chemically, it is thought that new vessels are able to secrete soluble factors that directly influence the differentiation of progenitor cells toward neuronal fate. The nature of this signaling remains still unclear and poorly investigated, but represents an essential step to ameliorate the therapeutic recovery from stroke and to elucidate the molecular mechanisms involved in tissue regeneration after an injury.

Regenerative medicine is the branch of science that exploits these biological mechanisms to replace and regenerate cells, tissues, or organs. It is a promising approach to dvelop treatments for degenerative disorders and shares many cellular and molecular processes with developmental biology. Therefore, understanding the molecular pathways at play during the development of the vascular and neural systems could lead to innovations and improved techniques in the field of regenerative medicine, especially as treatment for neurological disorders with a strong vascular component.^[^ ^7^, ^18^ ^]^

Studying similarities between the vascular and nervous systems, especially their molecular mechanisms, could provide valuable insight into processes important in their regeneration and possibly improve current treatments for neurovascular disorders. Indeed, research is usually focused on either vascular or nervous regeneration. Adopting a neurovascular perspective, by comparing processes involved in both systems, could improve the quality of the treatments offered. Comparing molecular processes known to be involved in both systems separately can provide insights into promising areas of future research. On top of that, development and regeneration share many mechanisms. Therefore, comparing signaling molecules involved in the development of the vascular and nervous systems and shedding light to those that they have in common can reveal processes with great potential, which have not yet been studied from a regenerative perspectivel.

Accordingly, this review discusses and compares processes involved in the development of the vascular and nervous systems, in order to provide an overview of the molecular mechanisms, which are most promising for the treatment of neurovascular disorders. The term “neural” will be used to define cells of the nervous system, namely neurons and glia, while “neuronal” solely refers to neurons.

## 2 Vascular System

### 2.1 Research History

Discovering and understanding the functioning of the vascular system was a major turning point in the history of biology, as it provided a new explanation for the way complex organisms were structured. Its study can be traced back to thousands of years, including an early distinction of arteries from veins, and studies on the anatomy of the heart. However, most of our current knowledge on the circulatory system has been acquired since the 19th century.^[^ ^19^ ^]^

Theodore Schwann described the endothelium for the first time in 1845, following his observations of cell nuclei along capillary walls.^[^ ^19^ ^]^ From then on, understanding molecular processes involved in vasculogenesis and the maintenance of the endothelium became a focus of scientific research. In 1951, the number of retinal capillaries was found to increase in hypoxic environments: this observation later led to the discovery of vascular endothelial growth factor (VEGF) in 1983 by Senger et al.,^[^ ^20^ ^]^ a guidance cue important in vascular development, whose concentrations increase in settings of low oxygen concentrations.^[^ ^19^ ^]^ It was first believed that signaling molecules playing an important role in vascular development were specific to said system, reinforcing the idea that VEGF was unique to the vasculature. However, it is now known that many molecular processes involved in the developing vascular system, including VEGF, have significant functions in other developing systems as well.

### 2.2 Mechanisms of Development at the Cellular Level

#### 2.2.1 Main Steps Leading to a Developed Vascular System

Prenatal vascular development starts when angioblast precursors in the embryo proper form and aggregate, creating blood islands in the visceral yolk sac.^[^ ^1^, ^21^ ^]^ Vasculogenesis then takes place aggregating blood islands into a primitive network of endothelial tubes.^[^ ^1^, ^21^, ^22^, ^23^, ^24^ ^]^ Angiogenesis is the development of mature vessels through complex molecular processes, and includes proliferation and remodeling of the initial network (Figure 1).^[^ ^1^, ^21^, ^22^, ^23^, ^24^, ^25^, ^26^ ^]^ Finally, differentiation of primitive blood vessels into arteries or veins depends both on intrinsic and extrinsic factors. For example, vessels can show a genetic predisposition for either phenotypes, but external factors such as haemodynamic forces can affect the outcome of blood vessel differentiation as well.^[^ ^27^, ^28^ ^]^ Haemodynamic factors include shear stress and blood pressure, perceived by endothelial cells through circumferential wall stress.

#### 2.2.2 Tip Cells

**Figure 2:**
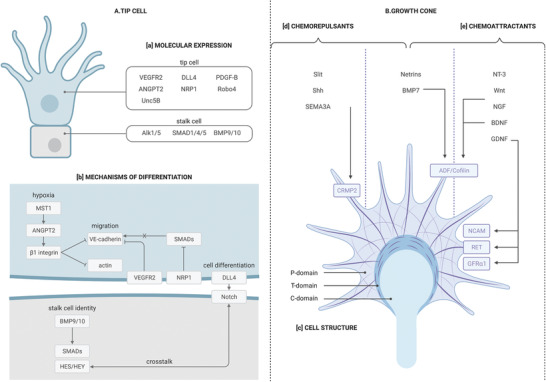
Cellular structure and behavior of tip cells and growth cones. A) Tip cells guide developing vascular cells, and are essential for the formation of blood vessels. a) Tip and stalk cells expressed different markers. Among others, VEGFR2, DLL4, and PDGF‐B can be found on tip cells, while Alk1/5, several SMADs, and BMP9/10 are associated to stalk cells. b) Molecular pathways at play in tip cell selection and maintenance of tip and stalk cell identity are known to involve hypoxia, and signaling molecules such as VE‐cadherins, VEGFR2, Notch, and BMPs. However, the complete pathway is still unclear. B) Growth cones guide developing axons. As opposed to tip cells which are separate from stalk cells, growth cones are an integral part of the developing neuron. c) Their structure consists of three parts. The P‐domain contains filopodia and lamellipodia, which respectively contribute to environment recognition and movement. The C‐domain contains organelles and a dense microtubule array. The T‐region is located between the previous two and contains myosin contractile structures, which regulate both actin and microtubules. Growth cones determine the direction in which neurons develop. They do so by sensing guidance molecules, which can either repel or attract them. Such cues are respectively known as d) chemorepulsive or e) chemoattractant. A few guidance molecules’ pathway of action has been elucidated, which involve CRMP2, ADF, and Cofilin, NCAM, RET, and GFR*α*1. Finally, some molecules are known to be involved in growth cone guidance, but their effect is still unclear. These include Netrins and BMP7.

Tip cells are specialized endothelial cells, leading developing blood vessels. They are motile, invasive, and polarized with a large number of filopodia.^[^ ^25^ ^]^ Stalk cells follow tip cells while proliferating, in order to elongate vessel sprouts and form lumens.^[^ ^25^, ^26^ ^]^ Endothelial cell phenotypes can be recognized through a series of markers, including VEGF Receptors. VEGFR2 and VEGFR1 are expressed on tip and stalk cells, respectively (**Figure** **2**).^[^ ^25^ ^]^

Tip cell shuffling refers to the ability of endothelial cells to continuously alternate between phenotypes. The endothelial cell pool is dynamic, allowing for a constant competition for the leading position and a more efficient branching of the vascular network. When stalk cells are no longer neighboring a tip cell, decreased inhibitory signals turn them into tip cells, ensuring that sprouting blood vessels will arise throughout the developing vascular system. Constant competition ensures that multiple tip cells within a localized area inhibit each other.^[^ ^25^ ^]^

Tip cells sense attractant and repulsive cues through their filopodia, in which adhesion and de‐adhesion processes are triggered, leading to movement. Molecular cues are therefore responsible for determining the direction taken by tip cells, and ultimately define the pattern along which vascular vessels are built.^[^ ^27^ ^]^

### 2.3 Mechanisms of Development at the Molecular Level

#### 2.3.1 VEGF/Placental Growth Factor (PIGF)

The VEGF family of growth factors is a group of homodimeric glycoproteins consisting of six ligands—VEGFA, VEGFB, VEGFC, VEGFD, VEGFE, and PIGF—and three receptors—VEGFR1, VEGFR2, and VEGFR3.^[^ ^1^, ^22^ ^]^

**Figure 3:**
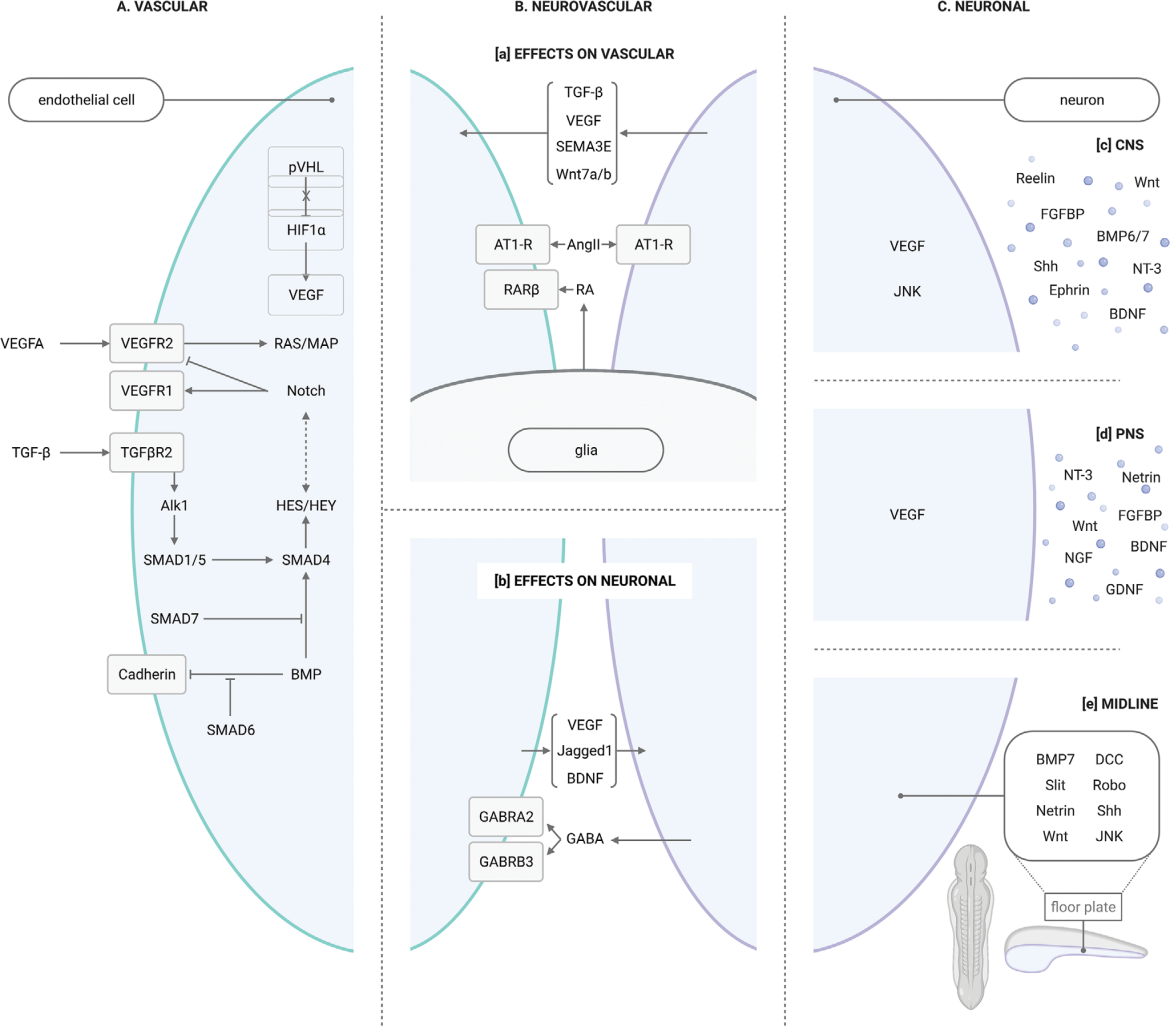
Signaling pathways within and between developing endothelial cells and neurons. A) Signaling pathways involved in the developing endothelial cell. Among many others, these include VEGF, Notch, TGF‐*β*, and BMP, which interact through SMADs and HES/HEY proteins. HIF1*α* increases VEGF levels under hypoxic conditions, indirectly promoting the development of new blood vessels in areas that are not yet vascularized. B) Developing blood vessels and nerves influence one another. a) Neurons influence growing endothelial cells through a series of mechanisms, which include TGF‐*β*, VEGF, SEMA3E, and Wnt7a/b. Glia also affect endothelial cells via RA. b) Endothelial cells, in turn, can also influence neuronal development. They can do so through VEGF, Jagged1, and BDNF. GABA's role in neuronal development is slightly different, and has been related to the development of a functional cortical structure. Endothelial cells across different brain areas express different GABA receptors, and guide developing neurons to their destination. Pial endothelial cells, found in the innermost layer of the meninges, express GABRA2, which guides the migration of superficial neurons, whereas periventricular endothelial cells express GABRB3, guiding inner deep neurons. C) Finally, molecular mechanisms involved in the development of the nervous system can be divided by the areas in which they act, namely the c) CNS and d) PNS. A few of the signaling molecules are found in both, including VEGF, NT‐3, Wnt, FGFBP, and BDNF. e) Midline crossing is an important process of nervous development that takes place in the CNS and involves commissural axons. Midline crossing is a process carried out by developing axons, when traveling from one hemisphere to the other, to connect contralateral brain areas. Once the midline has been crossed, it is extremely important for developing axons to synapse in the contralateral side, to prevent crossing multiple times. The process by which multiple‐crossing is avoided is complex and still being investigated. However, some key molecules have been identified, which include BMP7, Netrins, and Slit and Robo, among others. The floor plate is a structure that spans the anteroposterior axis of the developing nervous system and mediates midline crossing.

VEGFA, also known as vascular permeability factor (VPF), binds to both VEGFR1 and VEGFR2.^[^ ^1^, ^22^, ^24^ ^]^ VEGFR1 is thought to decrease the amount of VEGFA available to VEGFR2, as its affinity to the ligand is higher but the triggered kinase activity weaker (**Figure** **3**).^[^ ^26^ ^]^ Five isoforms of VEGFA, generated through alternative splicing of RNA, have been described. Their respective sequences are long of 121, 145, 165, 189, and 206 amino acids.^[^ ^22^ ^]^ The shortest isoform does not bind to heparin, which itself is bound to the extracellular matrix or cell surface, making VEGF121 the only diffusible isoform. On top of that, VEGF165 is the most physiologically relevant, while VEGF145 and VEGF206 are the least expressed.^[^ ^22^ ^]^ VEGFB, also known as VEGF‐related factor (VRF), binds to VEGFR1.^[^ ^1^, ^22^, ^24^ ^]^ Its gene expression was shown not to be induced by hypoxia, and it has been suggested to play a special role in muscle vascularization.^[^ ^1^, ^22^, ^24^ ^]^ VEGFC, also known as VEGF‐related protein, binds to VEGFR3,^[^ ^1^, ^22^, ^24^ ^]^ and is induced by proinflammatory cytokines.^[^ ^22^ ^]^ VEGFD, also known as C‐Fos‐Induced Growth Factor (FIGF), binds to VEGFR3,^[^ ^1^, ^22^, ^24^ ^]^ and is induced by the transcription factor C‐Fos.^[^ ^22^ ^]^ VEGFE is virally encoded,^[^ ^22^, ^24^ ^]^ and will therefore not be addressed further in this review. PIGF binds to VEGFR1,^[^ ^22^, ^24^ ^]^ and shares 46% of its amino acid sequence with VEGFA.^[^ ^22^ ^]^ PIGF homodimers and PIGF‐VEGF heterodimers are thought to be weak inducers of angiogenesis and vascular permeability, in comparison to VEGF homodimers.^[^ ^22^ ^]^

VEGFR1, also referred to as Flt1, triggers homodimerization, autophosphorylation, and activation of a downstream signaling cascade,^[^ ^1^ ^]^ which is thought to induce a weak kinase activity.^[^ ^26^ ^]^ Embryos lacking this receptor produced both functioning haematopoietic and endothelial cells.^[^ ^22^ ^]^ However, the formation of functioning blood vessels was defective and led to death.^[^ ^22^ ^]^ Therefore, VEGFR1 is thought not to be of importance during vasculogenesis, but its signaling has been suggested to regulate endothelial cell‐cell or cell–matrix interactions during vascular development.^[^ ^22^ ^]^ VEGFR2, also referred to as KDR or Flk1, causes homodimerization, autophosphorylation, and activation of a downstream RAS/MAP kinase signaling cascade (Figure 3).^[^ ^1^ ^]^ This receptor is thought to play a role in vasculogenesis, as its deficiency in embryos caused a decreased production of haematopoietic precursors and angioblasts, and death at approximately embryonic day 9.^[^ ^22^ ^]^ VEGFR3, also referred to as Flt4, is made of seven immunoglobulin‐like domains used for binding to the VEGF ligand and an intracellular kinase domain. It is believed to be present across the early vascular system during development, but is later restricted to the developing lymphatic system.^[^ ^1^ ^]^

VEGF is believed to act in response to hypoxia. Indeed, under normal conditions, a protein known as Von Hippel‐Lindau tumor suppressor (pVHL) ubiquitinates a subunit of the Hypoxia‐Inducible Factor (HIF1*α*), leading to its proteasome degradation. However, when oxygen concentrations are low, pVHL is blocked: HIF then translocates to the nucleus, binds to hypoxia‐response elements (HREs) and triggers the expression of a number of hypoxia‐responsive genes, including VEGF (Figure 3).^[^ ^1^ ^]^

VEGF is thought to play a role in tip cell selection. VEGFR2 and VEGFR1 are respectively expressed on tip and stalk cells, and endothelial cells with higher VEGFR2 and lower VEGFR1 levels therefore have increased chances of acquiring and maintaining the leading position in developing blood vessels.^[^ ^25^ ^]^ Notch signaling is believed to be involved in this process. Loss of a single allele of Notch ligand Delta‐Like 4 (DLL4) in mice resulted in a considerable increase in tip cell number.^[^ ^27^, ^29^ ^]^ Tip cells increase their expression of Notch ligands on their cell membranes, especially DLL4, which activate Notch receptors in adjacent stalk cells. When Notch ligand expression is suppressed, VEGFR1 transcription is induced and VEGFR2 expression is inhibited through Cardiovascular Helix‐Loop‐Helix Factor 1 (CHF1).^[^ ^24^, ^25^ ^]^ These events enhance stalk cell differentiation.^[^ ^27^ ^]^

Other functions of VEGF include attracting tip cells and enhancing vessel permeability. Depending on its isoform, VEGF can attract tip cells on a short or long‐range.^[^ ^27^ ^]^ VEGFA causes Src phosphorylation of VE‐cadherins in mature vessels, leading to weakened cell‐cell junctions and increased permeability.^[^ ^26^ ^]^

#### 2.3.2 Delta/Notch

The Notch signaling pathway is an important process in cell‐cell communication. It comprises five ligands—Delta‐like 1 (DLL1), DLL3, DLL4, Jagged1, and Jagged2—and four receptors—Notch1, Notch2, Notch3, Notch4. When a receptor is activated, its intracellular domain is proteolytically cleaved, a fragment of which binds to the transcription factor recombination signal binding protein for immunoglobulin kappa J region (RBPJ). Together, the fragment and RBPJ enter the nucleus and trigger the transcription of target genes, including HES/HEY, which regulate cellular differentiation and the fate of stem and progenitor cells.^[^ ^1^ ^]^

Delta and Notch molecules play a role in blood vessel differentiation into arteries and veins. Indeed, Notch1, Notch4, DLL4, Jagged1, and Jagged2 were shown to be expressed in arterial endothelium, while they were found at extremely low levels in veins (Figure 1).^[^ ^1^, ^27^ ^]^

The Notch pathway was also shown to play a role in vessel quiescence and stability. By reducing the VEGFR2 levels on endothelial cells and increasing their expression of VEGFR1, Notch lowers their responsiveness to VEGFA (Figure 3). Inhibition of DLL4/Notch signaling in adults has resulted in hyper‐proliferative vascular lesions in the liver and skin, endorsing that Notch signaling is necessary to maintain endothelial cell quiescence in adulthood.^[^ ^26^ ^]^

#### 2.3.3 Fibroblast Growth Factor (FGF)

FGF is a family of cell signaling proteins which, when binding to their corresponding receptors (FGFR), trigger the activation of the classical MAP kinase pathway, in turn regulating gene expression.^[^ ^30^ ^]^ Over twenty ligands—whose names range from FGF1 to FGF23—and their four receptors—FGFR1, FGFR2, FGFR3, and FGFR4—have been identified in humans.

FGFs were thought to support endothelial cell proliferation, migration, and differentiation.^[^ ^30^ ^]^ However, their function in angiogenesis is still controversial as contradicting results have been obtained in different studies. One study found that endothelial deletions of FGFR1 coupled with global deletions of FGFR3 in embryos resulted in defects in blood vessel development in the skin and retina.^[^ ^31^ ^]^ Another study found that mutants lacking either ligand or receptors did not present defective cardiovascular systems, while yet another found that receptor blocker injections resulted in considerable damage to the developing vascular system in mice.^[^ ^30^ ^]^

#### 2.3.4 Angiopoietin (ANGPT)/Tyrosine Kinase with Immunoglobulin‐Like and EGF‐Like Domains (TIE)

Four ligands—ANGPT1, ANGPT2, ANGPT3, and ANGPT4—signal through two tyrosine kinase receptors: TIE1 and TIE2.^[^ ^26^ ^]^ ANGPT2 and TIE2, also known as TEK, play a role in tip cell selection and vascular remodeling. ANGPT2 expression is thought to be induced by VEGFA and specific to tip cells, while TIE2 expression is considerably low in the leading cells.^[^ ^26^ ^]^ The absence of TIE2 receptors enhances the binding of ANGPT2 to *β*1 integrin, whose activation reduces cortical actin and weakens VE‐cadherin‐containing adherens junctions, promoting endothelial cell migration and sprouting angiogenesis (Figure 2).^[^ ^26^ ^]^ Hypoxia also upregulates ANGPT2 expression through activation of the kinase Mammalian Sterile 20‐Like Kinase 1 (MST1).^[^ ^1^ ^]^ ANGPT1 and ANGPT4 are believed to stimulate TIE2 autophosphorylation and its downstream pathways, while ANGPT2 and ANGPT3 downregulate them.^[^ ^1^ ^]^ Inhibition of TIE2 leads to unstable vessels, in which case VEGF is needed for successful remodeling. In a case where VEGF would not be present, the destabilized vessels would be dysfunctional and endothelial cells would apoptose, resulting in vessel regression.^[^ ^1^ ^]^

#### 2.3.5 Semaphorins (SEMA)/Plexins/Neuropilins (NRP)

Semaphorins are a family of ligands, classified into classes, depending on their structure and species of origin: 1 and 2 are found in invertebrates, while 3 to 7 belong to vertebrates.^[^ ^32^, ^33^ ^]^ However, the distinction has become ambiguous, as their presence is increasingly found across species.^[^ ^33^ ^]^ Their effects can be mediated through Plexins and Neuropilins. Plexins—Plexin‐A1, Plexin‐A2, Plexin‐A3, and Plexin‐A4—belong to the c‐Met family and can transactivate a very diverse collection of other receptor tyrosine kinases, including VEGFR2 and FGFR2.^[^ ^25^, ^34^ ^]^ Neuropilins—NRP1 and NRP2—can be activated by Class 3 Semaphorins.^[^ ^1^, ^34^ ^]^

Semaphorins have opposing effects on angiogenesis. Indeed, SEMA3A, SEMA3B, SEMA3D, and SEMA3F are thought to inhibit integrin function and thus result in anti‐angiogenic effects.^[^ ^25^ ^]^ SEMA3E has been shown to inhibit tumor angiogenesis, while SEMA4D, SEMA5A, and SEMA6A promote angiogenesis.^[^ ^25^ ^]^ SEMA3E has also been studied in relation to VEGF regulation, as part of a possible feedback mechanism.^[^ ^34^ ^]^

Neuropilins have been related to other functions in angiogenesis. NRP1 is thought to be specific to arterial endothelial cells, while NRP2 is present solely on veins (Figure 1).^[^ ^1^, ^27^ ^]^ Some VEGFA isoforms can bind to both NRP1 and NRP2.^[^ ^1^, ^22^, ^26^, ^35^ ^]^ NRP1 and NRP2 respectively interact with VEGFR2 and VEGFR3.^[^ ^24^ ^]^ Additionally, NRP1 is thought to promote tip cell selection by enhancing VEGFR2 signaling, as NRP1‐deficient endothelial cells show lower levels of VEGFR2 signaling.^[^ ^35^ ^]^ Finally, Neuropilin overexpression in transgenic embryos led to excess vascularisation in the developing brain.^[^ ^1^ ^]^

#### 2.3.6 Platelet‐Derived Growth Factor Subunit B (PDGF‐B)

PDGF‐B is a human protein whose functions range from regulating embryonic development and cell proliferation, to wound healing in adulthood. It has been associated to several functions in angiogenesis, including tip cell differentiation, response to haemodynamic factors, arterial differentiation, and regulation.^[^ ^36^ ^]^ Indeed, tip cells were shown to express higher levels of PDGF‐B than stalk cells (Figure 2).^[^ ^36^ ^]^ It is believed that PDGF‐B expression could be triggere increased in arterial walls, as big arteries are exposed to higher stress from blood flow.^[^ ^27^ ^]^ However, PDGF‐B expression in arterial endothelial cells was greater than in veins of equivalent wall thickness,^[^ ^36^ ^]^ suggesting that haemodynamic factors might only be part of the mechanism behind PDGF‐B‐related arterial differentiation. Finally, PDGF‐B has been shown to be involved in the regulation of pericyte recruitment, investment, and retention. The ligand, released from angiogenic endothelial cells, binds to its receptor PDGFRB expressed by developing pericytes.^[^ ^1^, ^36^, ^37^ ^]^

#### 2.3.7 Mothers against Decapentaplegic Homolog 6 (SMAD6)

SMAD proteins are the main signal transducers for receptors of the transforming growth factor beta (TGF‐*β*) superfamily.^[^ ^38^ ^]^ The acronym refers to homologous proteins: SMA stands for the “small” phenotype found in Caenorhabditis elegans, while MAD is derived from the “Mothers Against Decapentaplegic” family of genes found in Drosophila.^[^ ^39^ ^]^ Eight SMADs exist, whose names range from SMAD1 to SMAD8/9.^[^ ^40^ ^]^

Functions associated to SMAD6 include response to haemodynamic factors and blood vessel stabilization. SMAD6 expression was shown to be related to blood flow and could therefore respond to haemodynamic factors. It is mainly expressed in big arteries such as the dorsal aorta, vertebral arteries, branchial arch arteries, and the surface arteries of the brain, while being way less expressed in coronary arteries. SMAD6 is also thought to play a role in endothelial junction stabilization, as deficient mice died of vessel haemorrhage at late gestational stages and even early postnatal development.^[^ ^38^ ^]^

#### 2.3.8 TGF‐*β*

Named after its first member TGF‐*β*1, proteins from the TGF‐*β* superfamily interact with TGF‐*β* Receptors. Bone morphogenic proteins (BMP), which belong to the family, will be discussed separately. Three types of TGF‐*β* Receptors have been described: TGF*β*R1, TGF*β*R2, and TGF*β*R3, the first two being the main signal transducers.^[^ ^35^ ^]^

When bound to a ligand, TGF*β*R1 phosphorylates Activin Receptor‐Like Kinase 5 (Alk5). Alk5 then phosphorylates SMAD2/3, which in turn forms a complex with SMAD4 and translocates to the nucleus, where it activates the transcription of target genes. In contrast, TGF*β*R2 phosphorylates Alk1. Alk1, thought to be specific to endothelial cells, works through SMAD1/5, and also activates the transcription of target genes with SMAD4.^[^ ^1^, ^35^, ^36^ ^]^ SMAD4 enhances the transcription of HES/HEY genes, typically associated to Notch signaling, and through their cross‐talk, is believed to regulate VEGFR1, VEGFR2, and NRP1 levels (Figure 3).^[^ ^26^ ^]^ Additionally, TGF‐*β* was shown to regulate a vast array of genes involved in angiogenesis, including VEGF, ANGPT2, FGF, FGFR1, and Ephrin Type B Receptor 2 (EphB2). Alk1 regulated the transcription of Ephrin‐B1, integrin *α*E, ICAM‐1, and ICAM‐2, while Alk5 regulated Ephrin‐A1, Angiopoietin‐Like 4, and integrin *α*6.^[^ ^41^ ^]^

The signaling pathways resulting from the activation of TGF*β*R1 and TGF*β*R2 are not redundant, which can be explained by their distinct target genes. Indeed, mutations in either Alk1 or Alk5 caused vascular failure and were lethal to embryos. Loss of Alk1 signaling, for example, resulted in enlarged vessels and a failed distinction between arteries and veins.^[^ ^1^ ^]^ However, the respective functions of both signaling pathways are still controversial. The Alk5 pathway may be necessary to activate vasculogenesis, while Alk1 crucial during its resolution phase, mainly ensuring vessel stabilization through secretion of extracellular matrix, production of proteinase inhibitors, and recruitment of supporting cells.^[^ ^1^ ^]^ However, another study implied the opposite, suggesting Alk1 signaling leads to cell proliferation and migration, while Alk5 would be necessary for cell differentiation and extracellular matrix production.^[^ ^36^ ^]^ Overall, it is clear that TGF‐*β* plays a role in the regulation of angiogenesis, and could modulate a balance between endothelial proliferation and vessel stabilization.

Aside from endothelial proliferation, TGF‐*β* is believed to play a role in tip cell differentiation and in response to haemodynamic factors. Tip cells showed lower Alk1 levels than stalk cells, and endothelial cells deficient in Alk1, Alk5, SMAD1, SMAD4, or SMAD5 were shown to preferentially take the tip cell position.^[^ ^35^ ^]^ TGF‐*β* has been shown to contain a Shear‐Stress‐Responsive Element (SSRE) to which transcription factors can bind, and has therefore been suggested as a possible signaling mechanism participating in endothelial cell's sensitivity to haemodynamic forces.^[^ ^27^ ^]^ TGF‐*β* is believed to bind to NRP1 and compete with Semaphorins and VEGF in doing so. The complete mechanism has not been elucidated, but NRP1 could inhibit TGF‐*β* signaling.^[^ ^35^ ^]^

#### 2.3.9 BMP

BMPs are a family of over twenty proteins, nineteen of which belong to the TGF‐*β* superfamily. Similarly to its other ligands, BMPs trigger a signaling cascade which results in the translocation of SMAD4 into the cell nucleus, and the transcription of target genes.^[^ ^26^, ^38^ ^]^ Interestingly, BMP‐induced SMAD4 translocation enhances the transcription of HES genes, typically associated to Notch signaling.^[^ ^26^ ^]^ SMAD7 is thought to inhibit BMP‐induced SMAD signaling.^[^ ^38^ ^]^

BMPs are involved in multiple processes during angiogenesis, including roles in tip cell differentiation. BMP9/10 are thought to suppress the tip cell state and promote stalk cell identity through activation of SMADs and HES/HEY, allowing crosstalk with Notch signaling.^[^ ^26^, ^29^ ^]^ In tip cells, SMAD activation is suppressed by NRP1 (Figure 2).^[^ ^26^ ^]^

Several BMPs have been attributed opposing effects in angiogenesis, which could be explained by the different downstream mechanisms they trigger. BMP6 and BMP7, for instance, were shown to promote vessel sprouting, while BMP9 and BMP10 promote vessel quiescence and stability.^[^ ^26^, ^38^ ^]^ BMP2 was shown to promote vein‐specific sprouting in zebrafish independent of VEGFA.^[^ ^26^ ^]^

Finally, blood flow is thought to potentiate BMP9 signaling through Alk1 activation. Indeed, BMP9, Alk1, and SMAD4 mutants suffered from arteriovenous malformations as a result of heightened levels of PI3K/AKT signaling, which is activated downstream of VEGFA and repressed by BMP9 signaling.^[^ ^26^ ^]^

#### 2.3.10 Vascular Endothelial Cadherin (VE‐Cadherin)

VE‐cadherin, also known as Cluster of Differentiation 144 (CD144), is a type of cell adhesion molecule, especially important in endothelial cell adherens junctions.^[^ ^23^, ^38^ ^]^ VE‐cadherin plays many roles in angiogenesis, especially in relation to endothelial cell motility. In tip cells, VEGFR2 activates Src‐dependent VE‐cadherin phosphorylation, leading to its internalization, and ultimately weakens adherens junctions (Figure 2). This mechanism, which can be induced by BMP as well, allows for a VE‐cadherin turnover and endothelial cell migration, but has only been shown to be essential in some vessel types.^[^ ^26^, ^38^ ^]^ SMAD6 prevents VE‐cadherin internalization. Decreased levels of SMAD6 in endothelial cells resulted in increased VE‐cadherin internalization.^[^ ^38^ ^]^ Additionally, short intervals of shear stress resulted in the formation of VEGFR2, VE‐cadherin, and *β*‐catenin complexes, which seem to play a role in SSRE‐dependent gene transcription as a response to haemodynamic forces.^[^ ^27^ ^]^

#### 2.3.11 Slit/Roundabouts (Robo)

The functions of Slit—a family of extracellular matrix proteins—and Robo—their corresponding transmembrane receptors—in angiogenesis are still very controversial. Robo4, for example, has been shown to promote both cell migration and vessel stabilization. It has been reported to activate an anti‐angiogenic pathway resulting in a blockade of VEGF downstream signaling. An endothelial transmembrane protein, known as Unc5B and highly expressed on tip cells, has also been identified as a ligand of Robo4, and their interaction appears to result in stabilization of the vasculature. However, within tip cells, Robo4 is believed to stimulate filopodia formation, cell migration, and angiogenesis.^[^ ^25^ ^]^

#### 2.3.12 Retinoic Acid (RA)

RA is a hormone derived from Vitamin A, whose functions in embryonic development are well documented. RA modulates gene expression by entering the cell nucleus after binding to Retinoic Acid Receptors—RAR*α*, RAR*β*, RAR*γ*—and Retinoid X Receptors—RXR*α*, RXR*β*, RXR*γ*. Regarding vascular development, RA knockout in mouse embryos resulted in a primitive yolk sac vasculature, which failed to remodel, and an incomplete smooth muscle cell recruitment to large vessels.^[^ ^31^ ^]^

#### 2.3.13 COUP Transcription Factor 2 (Coup‐TFII)

Coup‐TFII, also known as Nuclear Receptor Subfamily 2 Group F Member 2 (NR2F2), is a receptor believed to be activated by retinoic acid. Its function in angiogenesis seems to be focused on vessel differentiation between arteries and veins. Indeed, through the inhibition of NRP1 and Notch signaling, Coup‐TFII enhances the expression of venous markers such as EphB4. Coup‐TFII deletion in venous endothelial cells in mutant mice allowed them to acquire arterial markers such as EphrinB2. However, the transition was not complete, suggesting the involvement of other signaling mechanisms.^[^ ^27^ ^]^

#### 2.3.14 Sonic Hedgehog (Shh)

Shh is a protein which, when internalized, causes transcription factor Gli2 and Gli3 to enter the nucleus and modulate gene expression. Downstream target genes of the pathway include Gli1, Bcl2, and N‐myc, which are respectively involved in cell fate proliferation and determination, apoptosis regulation, and prenatal brain development. Its various key roles in development are well‐documented. However, in angiogenesis, due to contradicting results, it has not been established whether Shh promotes vessel integrity and quiescence or destabilizes vessels and promotes angiogenesis. Overall, the protein has been suggested to play a role in cell differentiation, vessel branching, arterial differentiation, and pericyte recruitment.^[^ ^42^ ^]^

#### 2.3.15 Erythropoietin‐Producing Hepatocellular Carcinoma Receptors (EphR)/Eph Receptor‐Interacting Signals (Ephrins)

Ephrins bind to Eph receptors. Two subtypes of Ephrins have been described—Ephrin‐As and Ephrin‐Bs. Ephrins bind to two receptor groups—EphA and EphB—all of which are tyrosine kinases. Eight different EphA exist, named EphA1 to EphA8, while the EphB group contains five molecules: EphB1 to EphB4, and EphB6.^[^ ^1^, ^32^ ^]^ EphA4 is the only EphA receptor able to bind to Ephrin‐Bs.^[^ ^1^ ^]^ EphrinB2 and EphB4 are respectively expressed in arterial and venous endothelial cells (Figure 1).^[^ ^1^, ^27^ ^]^ This distinction interestingly arises prior to the development of a functional circulation, suggesting the presence of an intrinsic preference during vessel differentiation.^[^ ^1^ ^]^ Additionally, Ephrins seem to be involved in the transduction of haemodynamic factors into the growth of lumen diameter.^[^ ^27^ ^]^

### 2.4 Mechanisms of Regeneration in Adulthood

#### 2.4.1 Mechanisms at the Cellular Level

Angiogenesis can be initiated during adulthood, and can be observed in diseases that increase tissue‐specific metabolic demand.^[^ ^26^ ^]^ Endothelial cells do not work alone during regenerative angiogenesis, as they are supported by myeloid cells and pericytes. Myeloid cells degrade the extracellular matrix and secrete vasoactive molecules and other growth factors.^[^ ^27^ ^]^ Studies have shown that pericytes are dominantly quiescent in established blood vessel networks, but can proliferate and differentiate into progenitor cells, when said network is being remodeled.^[^ ^37^ ^]^ They have been shown to differentiate into a vast array of lineages, including those of mesenchymal and neural stem cells.^[^ ^36^, ^37^ ^]^ Interestingly, in the adult brain, pericytes have been found directly adjacent to the tip cells of elongating blood vessels, suggesting a crosstalk between the two cell types in the CNS.^[^ ^43^ ^]^

#### 2.4.2 Mechanisms at the Molecular Level

VEGF and Notch have been shown to play a role in adult angiogenesis. VEGF165 induced cellular differentiation: Human multipotent adult progenitor cells (hMAPCs) differentiated into both arterial and venous endothelial cells when exposed to VEGF165, whereas AC133 cells solely differentiated into a venous phenotype.^[^ ^27^ ^]^ AC133 is a marker of hematopoietic stem and progenitor cells, derived from human foetal liver, bone marrow, and blood.^[^ ^44^ ^]^ The difference in response to VEGF165 was attributed to expression of Notch1 and Notch3.^[^ ^27^ ^]^ On top of Notch, HIF1*α* is believed to be involved in adult angiogenesis, and could trigger VEGF in settings of low oxygen levels.^[^ ^27^ ^]^

FGF has been involved in repair‐associated angiogenesis as well, and has shown positive results in vessel formation in models of ischaemia. Administration of FGF2 protein, FGF1‐encoding DNA, FGF2‐encoding adenoviruses, and FGF2 bioreactors resulted in increased blood vessel formation. However, results were affected by the administration route: intra‐venous administration was less effective than an equivalent intra‐arterial method.^[^ ^30^ ^]^

Hh signaling, whose activation is impaired in aging, is believed to be involved in adult angiogenesis. Administration of Hh ligands increased capillary density and promoted muscle perfusion in aged mice. However, Shh‐induced angiogenesis has been suggested not to involve Hh signaling, but instead rely on increased levels of VEGFA.^[^ ^42^ ^]^

EphrinB2 has also been related to improved angiogenesis, following ischaemic damage. Indeed, its levels are naturally upregulated in the arterial vasculature as part of the response to tissue ischaemia, and artificial administration resulted in enhanced angiogenesis. Here, EphrinB2 is believed to act through EphB4.^[^ ^27^ ^]^

### 2.5 Summary

Prenatal vascular development starts with the formation of blood islands,^[^ ^21^ ^]^ which then elongate and form endothelial tubes through vasculogenesis.^[^ ^1^, ^21^, ^22^, ^23^, ^24^ ^]^ Angiogenesis then turns them into mature blood vessels.^[^ ^1^, ^21^, ^22^, ^23^, ^24^, ^25^, ^26^ ^]^ Finally, vessels differentiate into arteries and veins (Figure 1).^[^ ^1^, ^21^, ^22^, ^23^, ^24^, ^25^, ^26^ ^]^ Several molecules of the Notch pathway, as well as SMAD6, NRPs, EphrinB2, and EphB4 are differentially expressed on arterial and venous cells.^[^ ^1^, ^27^, ^38^ ^]^ During vascular development, blood vessels contain two types of endothelial cells: tip and stalk cells. They can be distinguished by their morphology, as tip cells contain filopodia that sense their environment and aid movement.^[^ ^25^ ^]^ The two cell types also express different molecular markers, including VEGFR2, PDGF‐B, ANGPT2, and several BMPs (Figure 2).^[^ ^25^, ^26^, ^36^ ^]^ Tip cells can only be found at the start of growing vessels, and guide their development, while the tube itself is made of stalk cells.^[^ ^26^ ^]^ The processes through which tip cells recognize the direction they need to follow are not fully understood yet, but several guidance cues have been identified, the main one being VEGF.^[^ ^27^ ^]^ VEGF levels increase under hypoxic conditions, which drives developing blood vessels to areas which need vascularization (Figure 3).^[^ ^1^ ^]^ On top of that, blood vessels have to maintain a precise balance between endothelial proliferation and vessel stabilization. Although the mechanisms behind this process probably involve complex molecular pathways and are still unclear, it seems TGF‐*β* and BMPs could be involved.^[^ ^1^, ^32^ ^]^ Finally, development of the vascular network depends on the recruitment of supporting cells such as pericytes and smooth muscle cells, which are attracted via signaling molecules such as PDGF‐B, TGF‐*β*, RA, and Shh.^[^ ^1^, ^31^, ^37^, ^42^ ^]^ With regards to regeneration in adulthood, angiogenesis can be triggered after damage has occurred and involves cells such as myeloid cells and pericytes.^[^ ^26^ ^]^ Myeloid cells degrade extracellular matrix, allowing growing vessels to expand, and secrete vasoactive molecules and growth factors.^[^ ^27^ ^]^ Pericytes can differentiate into a vast array of progenitor cells, which include mesenchymal stem cells.^[^ ^36^ ^]^ Signaling molecules which have been shown to be relevant in vascular regeneration include VEGF, FGF, Hh signaling, and Ephrins.^[^ ^27^, ^30^, ^42^ ^]^ Future research is needed to confirm whether other signaling molecules at play during development also act during regeneration.

## 3 Neuronal System

### 3.1 Research History

The history of research in the field of developmental neurosciences cannot be discussed without mentioning Santiago Ramón y Cajal, a Spanish neuroscientist who won the Nobel Prize in Physiology and Medicine in the year 1906, together with the Italian biologist Camillo Golgi.^[^ ^45^ ^]^ His most relevant work in the context of this review involves the discovery of growth cones, which he described for the first time in 1890.^[^ ^5^, ^45^ ^]^ Supposedly inspired by the way leucocytes are attracted toward bacteria, through gradients of substances the latter produce, he suggested growth cones could guide axonal growth in response to molecular attractants.^[^ ^45^ ^]^ Once the existence of growth cones had been established, the next logical step was to understand which molecules were at play in their guidance, and how they each affected the trajectory of axonal growth. Currently, several chemoattractants and chemorepellent molecules have been identified, yet some of their mechanisms and interactions are not fully understood.^[^ ^33^, ^45^ ^]^

Further investigation revealed that guidance cues can be involved in multiple different developing systems, and are not necessarily specific to a single one. For example, most of the signaling molecules which guide growing axons, influence tip cells as well.^[^ ^19^ ^]^ This theory, however, cannot be assigned to a single scientist. A variety of studies and research teams, which are often each focused on single signaling molecules, have confirmed the distinct roles of specific mechanisms, together accumulating a growing amount of evidence.

### 3.2 Mechanisms of Development at the Cellular Level

#### 3.2.1 Neurulation and Neuronal Migration Methods

The nervous system starts its development when the primitive pit of the mesoderm becomes the notochord, which further turns into the primitive streak. The ectoderm surrounding the notochord becomes the neuroectoderm, and folds, forming the neural tube and neural crest, from which the CNS and PNS will respectively arise (Figure 1).^[^ ^2^, ^3^ ^]^

Migration of recently developed neurons across the developing nervous system depends on the area and its structure. In areas of the CNS organized in layers, such as the cerebral cortex, hippocampus, or cerebellum, young neurons crawl along radial glia with the help of surface adhesion molecules, extracellular matrix adhesion molecules, and associated signal transduction molecules.^[^ ^2^ ^]^ Such molecules—which include ɑv‐integrin, laminin, fibronectin, and ngcamL1 on glia, and CdK5/P35, neuroregulin, NMDA‐R1, ɑ3*β*1‐integrin on neurons—are often found to play a part in axonal growth and guidance.^[^ ^2^ ^]^ In areas of the CNS organized in nuclei, neural migration happens without the intervention of glial cells.^[^ ^2^ ^]^ In the PNS, adhesion molecules in the extracellular matrix, and molecules on the surfaces of cells of the surrounding embryonic tissues, guide new neurons.^[^ ^2^ ^]^ Sensory and sympathetic neurons respectively arise from dorsal root ganglia and sympathetic ganglia, while motor neurons from the ventral spinal cord.^[^ ^46^ ^]^

Long‐distance neural migration has been described in some brain areas and is still under investigation. Research has shown that neurons sometimes settle in different areas to the ones they originate from. For example, most neurons in the pulvinar thalamic nucleus arise in the diencephalon, but some migrate from the telencephalon. A consistent proportion of neurons from the cerebral cortex have a tangential rather than radial route during their migration, and many neurons producing gamma‐aminobutyric acid (GABA) in the cortex and olfactory bulb, as well as oligodendrocytes throughout the forebrain, were shown to arise from the ventral forebrain. The reason for such long‐distance neural movements is still unknown, but brain function has been speculated to benefit from an increased variety in neuronal origins.^[^ ^2^ ^]^

#### 3.2.2 Synapse Formation, Selective Elimination, and Importance of Glia

Synapse formation during prenatal development can reach a rate of ≈42.3 million synapses per minute,^[^ ^21^ ^]^ and is followed by selective elimination. Essentially, an excess of axons is created, and those who do not form synapses are removed. This process ensures the creation of an effective network of synapses.^[^ ^5^ ^]^ Neurotrophic factors and cell‐intrinsic mechanisms, involving the caspase cascade and ubiquitin‐proteasome systems in neurons, are responsible for keeping innervated neurons alive, thus indirectly leading to the deletion of the others. This process is necessary for the maturation of circuits in the nervous system.^[^ ^47^ ^]^

Glial cells are essential in synapse elimination. Terminal Schwann cells are non‐myelinating and contribute to synapse deletion in neuromuscular junctions, where they form processes between axon terminals and muscle fibers, tightly against the plaque of acetylcholine receptors, blocking their communication.^[^ ^47^ ^]^ Once a synapse has been eliminated, they phagocytose its remains.^[^ ^47^ ^]^ Astrocytes can phagocytose synaptic material as well, by impairing the Calcium signaling necessary for neuronal functioning, supposedly through a reduced ATP release.^[^ ^47^ ^]^ Microglia are capable of sensing neuronal activity and removing neurons whose inputs are weaker, also through phagocytosis.^[^ ^47^ ^]^ Molecular signals from apoptotic neurons, including phosphatidylserine and complement cascade components, can be recognized by microglia, as triggers of phagocytosis. C3‐tagged synapses, for example, will be recognized by Microglial Complement Receptor 3.^[^ ^48^ ^]^ Microglia are also believed to modulate the apoptotic activity of developing neurons through the release of other molecular signals, including a combination of cytokines, such as IL‐1*β*, IL‐6, TNF*α*, and IFN*γ*, or Reactive Oxygen Species (ROS).^[^ ^48^ ^]^

#### 3.2.3 Growth Cone

The growth cone is a structure present at the tips of developing axons and dendrites. It has an extremely specific morphology, is highly mobile, and navigates through the developing nervous system by sensing molecular guidance cues.^[^ ^5^, ^32^, ^33^ ^]^ Growth cones contain mitochondria, ribosomes, branched membranous reticulum, lysosomes, cytoskeleton, membranous disks, and vacuoles.^[^ ^49^ ^]^ Their motility is ensured by highly dynamic cytoskeletal elements, such as actin and tubulin filaments, and proteins that modulate them.^[^ ^33^ ^]^ Its cellular structure can be divided into three sections. The P‐domain contains filopodia and lamellipodia, which respectively contribute to environment recognition, and movement. The C‐domain contains organelles and a dense microtubule array. The T‐region is located between the previous two and contains myosin contractile structures, which regulate both actin and microtubules (Figure 2).^[^ ^32^ ^]^

### 3.3 Mechanisms of Development at the Molecular Level

#### 3.3.1 VEGF/Hypoxia‐Inducible Factor (HIF1*α*)

Aside from its role in vascular development, VEGF has been shown to participate in the development of the nervous system (Figure 3).^[^ ^50^, ^51^ ^]^ In the CNS, VEGF promotes axonal and dendrite growth, as shown in retinal explants,^[^ ^52^ ^]^ olfactory bulb interneurons,^[^ ^53^ ^]^ and hippocampal and cortical neurons in vivo and in vitro.^[^ ^54^, ^55^, ^56^ ^]^ In zebrafish, NRP1 silencing resulted in abnormal branching and migration of motor neurons, suggesting a role for VEGF in axonal migration.^[^ ^57^ ^]^ Within the PNS, VEGF has been shown to act in multiple processes. Among others, it promotes axonal growth in dorsal root ganglia, by attracting growth cones, as well as modulating their velocity and size.^[^ ^58^, ^59^ ^]^ VEGF is also believed to promote Schwann cell proliferation and migration through VEGFR2.^[^ ^60^ ^]^

Finally, HIF1*α* has been discussed in the context of neural development. HIF1*α* knockouts in mice resulted in impaired survival and proliferation of preganglionic and postganglionic neurons of the sympathetic PNS, respectively.^[^ ^61^ ^]^

#### 3.3.2 FGF Binding Proteins (FGFBP)

FGFBPs have been studied in relation to the developing nervous system. In mice CNS, FGFBP3 was found to be expressed at higher levels during development than in adulthood. The same study found that FGFBP3 was mainly expressed by neurons. FGFBPs are believed to be important in the wiring of the brain and ensuring a correct functioning of cortical functions. However, the specific role and mechanism of action of these molecules in the developing CNS are not fully understood.^[^ ^62^ ^]^ In the PNS, the function of FGFBP1 has been studied in relation to the development of neuromuscular junctions. FGF7, FGF10, and FGF22 enhance the maturation of presynaptic regions by binding to FGFBP1 and acting through FGFR2, which is located on the presynaptic membrane and can promote the clustering of synaptic vesicles.^[^ ^62^ ^]^

#### 3.3.3 Semaphorins (SEMA)/Plexins/Neuropilins (NRP)

Semaphorins were shown to be implicated in several aspects of neural development, especially in axonal growth and guidance. SEMA3E, for example, mediates axon growth by interacting with VEGFR2 and NRP1, while multiple Semaphorins act as chemoattractants.^[^ ^34^ ^]^ SEMA3A, however, is believed to be responsible for a mechanism known as surround repulsion. In the surroundings of the tissue from which it is secreted, SEMA3A causes growth cones to collapse through an intracellular signaling protein, known as Collapsin Response Mediator Protein‐2 (CRMP2), thus inhibiting neuronal grow and innervation in that area (Figure 2).^[^ ^32^, ^33^ ^]^ However, depending on intracellular cyclic nucleotide levels, SEMA3A is believed to sometimes act as a chemoattractant.^[^ ^63^ ^]^

Guidance of developing Gonadotropin‐Releasing Hormone (GnRH) neurons has shown a special affinity for Semaphorins. Indeed, a lack of SEMA3A in mice resulted in defective GnRH neuron migration, shown by the formation of clusters in the nasal compartment, which were unable to reach the forebrain.^[^ ^34^ ^]^ The mechanism behind this process is thought to involve NRP1 and NRP2.^[^ ^34^ ^]^ Other roles assigned to Semaphorins include synapse formation through cytoskeleton reorganization of the axonal growth cone, neuronal apoptosis, and dendrite growth.^[^ ^64^ ^]^ In the PNS, SEMA3A was shown to play a role in growth cone guidance, through NRP1, as both SEMA3A and NRP1 mutant mice embryos showed abnormal morphogenesis of sympathetic neurons.^[^ ^65^ ^]^ Semaphorins are believed to modulate somite‐induced neural crest cell migration: secreted by the caudal end of somites, they act in a chemorepulsive manner.^[^ ^66^ ^]^

#### 3.3.4 BMP

BMPs are heavily involved in neural development, and their role starts in the differentiating neural plate. BMP presence elsewhere results in epidermal‐like differentiation. However, at the neural plate, Noggin and Chordin inhibit BMP signaling, inducing neural development.^[^ ^2^ ^]^ Aside from being inhibited during the early stages of neural development, several BMPs—especially BMP6 and BMP7—have been shown to be actively involved in the latter stages. BMP6 and BMP7 both control the induction and differentiation of dorsal interneurons in the spinal cord.^[^ ^32^ ^]^ BMP7 regulates growth cone motility through ADF/Cofilin, an actin‐binding protein, which is responsible for disassembling actin.^[^ ^32^ ^]^ However, it is not yet clear whether BMP7 attracts or repels growth cones. Indeed, it was found to repel commissural axons away from the dorsal root in vitro, but also to both attract and repel spinal neuron in a time‐dependent manner in frogs, providing evidence for a dual role of BMP7 in axon guidance (Figure 2).^[^ ^32^ ^]^

#### 3.3.5 Slit/Roundabouts (Robo)

Slit was shown to act as a repulsive cue in neural development (Figure 2), especially in that of retinal axons at the optic chiasm and neuronal precursors migrating to the olfactory bulb, as well as a branching factor in sensory axons and cortical dendrites.^[^ ^33^ ^]^ Slit and Robo, together, are mainly known for controlling midline crossing in developing neurons.^[^ ^33^ ^]^ Midline crossing is a process carried out by developing axons, when traveling from one hemisphere to the other, to connect contralateral brain areas. Once the midline has been crossed, it is extremely important for developing axons to synapse in the contralateral side to prevent crossing multiple times. The process by which multiple‐crossing is avoided is complex and still being investigated. However, some key molecules have been identified. In Drosophila, a protein known as Commissureless (comm) was shown to modulate Robo expression, ensuring increased levels after crossing and thus impeding axons to cross more than once.^[^ ^33^ ^]^ In humans, Robo3 isoforms are believed to play a similar role as comm: Robo3.1 and Robo3.2 are expressed in axons before and after crossing the midline, respectively. Robo3.1 inhibits the repulsive action of Robo1 and 2, while Robo3.2 enhances them.^[^ ^33^ ^]^ Differential Robo combinations in developing axons are also thought to control the end position of axons, once they have crossed the midline.^[^ ^33^ ^]^

Slit proteins interact with the Netrin pathway to control midline crossing, however the process is still unclear. Several hypotheses have been built. Robo levels were suggested to increase in axons that have crossed the midline, enhancing their responsiveness to Slit, and decreasing Deleted in Colorectal Cancer (DCC) signaling through Netrin‐1.^[^ ^33^ ^]^ DCC can act both as a chemoattractant and repellent in axonal guidance.^[^ ^67^ ^]^ Another interpretation focused on Netrin, and its downstream pathway being inhibited. Slit and Robo form a complex after midline crossing and bind to DCC, which is therefore unable to receive Netrin and mediate its effects.^[^ ^5^ ^]^

#### 3.3.6 Sonic Hedgehog (Shh)

Shh is extremely important in the developing nervous system. Its roles include regulating the development of motor neurons in the spinal cord,^[^ ^2^ ^]^ patterning the ventral spinal cord, guiding retinal ganglion cell neurons and olfactory sensory neurons, and guiding the migration of commissural axons.^[^ ^32^ ^]^ The process through which Shh is involved in the guidance of commissural axons is extremely complex, presumably involving a delicate cross‐talk with the Wnt signaling pathways.^[^ ^32^ ^]^ Overall, Shh seems to repel developing neurons (Figure 2).^[^ ^32^ ^]^

#### 3.3.7 Erythropoietin‐Producing Hepatocellular Carcinoma Receptors (EphR)/Eph Receptor‐Interacting Signals (Ephrins)

Ephrins were shown to have multiple functions in the developing nervous system, which include modulating the topographic mapping of the tectum. Ephrin‐A and Ephrin‐B molecules are believed to be responsible for modulating the anterior‐posterior and dorsal‐ventral axis, respectively.^[^ ^33^ ^]^ Aside from their role in topographical mapping, Ephrins can act as short‐range chemoattractants and repellents,^[^ ^32^, ^33^ ^]^ and are important in branching axons, regulating dendritic morphology, and synapse formation.^[^ ^33^ ^]^ EphA also plays a role in commissural axon guidance in the chicken hindbrain, while EphB regulates axonal guidance in the ventral midline and retina of mice.^[^ ^32^ ^]^

#### 3.3.8 Wingless/Int‐1 (Wnt)

Wnt proteins can act through two main signaling pathways: canonical and non‐canonical. Canonical pathways are *β*‐catenin‐dependent: Wnt proteins bind to Frizzled receptors and enter their target cell. Once inside, Wnt causes the degradation of a *β*‐catenin‐containing cytoplasmic protein complex, thus freeing *β*‐catenin, which enters the nucleus and modulates gene expression. Noncanonical pathways are *β*‐catenin‐independent, and triggered by the binding of Wnt to either Frizzled receptors or the Ryk/Derailed Receptor Tyrosine Kinase.^[^ ^32^ ^]^

Wnt proteins regulate a vast array of processes in the developing nervous system, including growth cone guidance, and neural differentiation of cerebellar granule cells and forebrain neurons.^[^ ^2^ ^]^ Wnt signaling also participates early on, promoting the specification of the PNS.^[^ ^66^ ^]^ The canonical and noncanonical pathways were suggested to participate in distinct processes, respectively being involved in neuronal regeneration following an injury, and axonal guidance during development.^[^ ^32^ ^]^ However, new evidence suggests both pathways might be more closely related than previously thought, and especially highlights the involvement of the *β*‐catenin‐dependent pathway in development. Indeed, studies have shown the importance of the canonical pathway during development: in cellular migration, proliferation, and myelination by both Schwann cells and oligodendrocytes.^[^ ^68^ ^]^ Wnt, independently of its noncanonical pathways, was found to attract midline‐crossing axons in neuronal development (Figure 2).^[^ ^32^ ^]^

#### 3.3.9 Netrins

Netrins are secreted proteins, involved in axonal guidance during neural development. A study conducted on mice reported that Netrin‐1 loss of function resulted in increased severe axon guidance defects, and earlier death.^[^ ^69^ ^]^

Netrins are of special importance in commissural axons and midline crossing through DCC,^[^ ^5^, ^70^, ^71^ ^]^ as Netrin‐1 null mice showed a considerable decrease in commissural axons successfully crossing the midline.^[^ ^72^ ^]^ Netrin‐1 has been shown, by several studies, to act both as a chemoattractant and repellent in commissural axons,^[^ ^5^, ^33^, ^70^ ^]^ and developing axons in general (Figure 2).^[^ ^32^ ^]^ For example, Netrin‐1 acted as a chemoattractant for ventrally directed commissural axons, while it was a chemorepellent for trochlear motor neurons.^[^ ^32^ ^]^ Experiments on Drosophila and rodents suggested that the distinction might be due to differential cytosolic cAMP‐dependent activity.^[^ ^32^ ^]^ A study examining the relationship between Phospholipase C Gamma1 (PLC*γ*1) and Netrins in the developing brain concluded that Netrin‐1 activated PLC*γ*1 through Src kinases, ultimately inducing actin cytoskeleton rearrangement.^[^ ^73^ ^]^ PLC*γ*1 knockout and deficient mice respectively showed abnormal axon guidance in dorsal parts of the mesencephalon during embryogenesis, and structural alterations in the corpus callosum, substantia innominate, and olfactory tubercle in adulthood.^[^ ^73^ ^]^ Finally, Netrins’ role in the development of the PNS was studied in Drosophila. Netrin‐lacking mutants showed defects in motor axon projections, suggesting an important role for Netrins in guiding motor neurons to their target muscles.^[^ ^74^ ^]^

#### 3.3.10 Nerve Growth Factor (NGF)

NGF is believed to sustain innervated sensory and sympathetic neurons during selective synapse elimination. Indeed, neurons that have reached their target become NGF‐dependent, and compete for survival.^[^ ^5^ ^]^ Additionally, NGF has been shown to modulate local chemoattraction.^[^ ^75^ ^]^ This process could also be observed in cases of re‐innervation: denervated tissue secretes NGF, attracting new developing axons.^[^ ^5^ ^]^ NGF is believed to act through Tropomyosin‐Receptor Kinase A (TrkA) and p75 Neurotrophin receptor (p75NTR) (Figure 2).^[^ ^32^, ^68^ ^]^ Other molecules mentioned as downstream effects of NGF's actions include ADF/Cofilin, in a similar fashion to BMP7,^[^ ^32^ ^]^ and myosin II, which could stimulate axon outgrowth.^[^ ^76^ ^]^

#### 3.3.11 Reelin

Reelin controls cellular migration and positioning in the developing cortex. The extracellular glycoprotein binds to Very Low‐Density Lipoprotein Receptor (VLDLR) and Apolipoprotein E Receptor 2 (ApoER2), triggering oligomerization of lipoprotein receptors, and ultimately leading to Domain Of Disabled‐1 (Dab1) phosphorylation by Src kinases. Reelin is secreted by Cajal‐Retzius neurons,^[^ ^77^ ^]^ which are located in the outermost layer of the developing brain, known as marginal zone, and disappear once neuronal migration is completed. It acts as a gradient and signals radially migrating neuron's final destination, thus defining cortical layers.^[^ ^77^ ^]^

#### 3.3.12 C‐Jun NH2‐Terminal Kinases (JNKs)

JNKs are Mitogen‐Activated Protein Kinases (MAPKs), and thus signal intracellularly. They are involved in neuronal differentiation, guidance of commissural axons, and dendrite and axon formation. By targeting chromatin modifiers, JNKs are indirectly able to modulate gene transcription through a regulation of histone phosphorylation and acetylation. In spinal cord neurons, target genes include transcription factors ATF‐2, Elk1, and NFAT4, as well as c‐Jun and p53, which are involved in cell cycle progression. Netrin increases JNK1 to induce midline crossing in commissural axons.^[^ ^78^ ^]^

#### 3.3.13 Neural Cell Adhesion Molecules (NCAMs)

NCAMs are cell surface glycoproteins, which in flies and mice, allow multiple axons to adhere to each other, in order to leave bundles and explore new paths together. Polysialic Acid (PSA) is supposedly necessary for this process. PSA, which has also been suggested to be specific to motor neurons, is a glycosylation on the surface of NCAMs.^[^ ^32^ ^]^

#### 3.3.14 Neurotrophin‐3 (NT‐3)

NT‐3 binds to Tyrosine Kinase Receptor C (TrkC), and acts as a chemoattractant, both during development and regeneration. It is known to play an important role both in the CNS and PNS. In rats, locally applied NT‐3 attracted Corticospinal Tracts (CSTs) to their target areas in the spinal grey matter.^[^ ^32^, ^68^ ^]^ Studies on the PNS, some of which performed in vivo, have shown that inhibiting NT‐3 signaling results in loss of sympathetic neurons.^[^ ^79^, ^80^, ^81^ ^]^ In the enteric nervous system, NT‐3 was found to act directly on precursor cells, promoting their survival and differentiation, and its offset is believed to be BMP2 and BMP4‐dependent.^[^ ^82^ ^]^

#### 3.3.15 Brain‐Derived Neurotrophic Factor (BDNF)

Initially, BDNF and its signaling through Tyrosine Kinase Receptor B (TrkB),^[^ ^68^ ^]^ were thought to be involved solely in the innervation of sensory neurons.^[^ ^32^ ^]^ For example, it had been shown to participate in the formation of the dorsal root ganglia.^[^ ^83^ ^]^ However, it is now known to play a role in a vast array of processes within neural development, including outside of the sensory PNS. Its signaling pathway, with a focus on Ca^2+^, has been studied in motor neurons.^[^ ^84^ ^]^ BDNF promotes neuronal survival and differentiation, as well as synapse formation.^[^ ^32^, ^68^ ^]^ BDNF also promotes axon guidance, by modulating growth cone motility through ADF/Cofilin, similarly to BMP7 (Figure 2).^[^ ^32^ ^]^ Within growth cones, a study using immunohistochemistry on rat dorsal root ganglion neurons showed an increase in protein synthesis related to BDNF activity.^[^ ^85^ ^]^ In chick embryos, BDNF was shown to increase growth cone motility by stimulating filopodial number and length.^[^ ^32^ ^]^

#### 3.3.16 Glia‐Derived Neurotrophic Factor (GDNF)

GDNF acts as a chemoattractant. It is believed to signal through GDNF Family Receptor Alpha‐1 (GFR*α*1), RET—a transmembrane tyrosine kinase receptor—and NCAMs (Figure 2).^[^ ^32^, ^68^ ^]^ Decreased GDNF levels were observed in patients suffering from chronic denervation, indicating a possibility for treatments involving GDNF.^[^ ^32^ ^]^ GDNF presence, in cultured porcine dorsal root ganglia neurons, resulted in neurite arborization and extension.^[^ ^86^ ^]^ Moreover, the SEMA3A‐mediated surround repulsion—a process that causes growth cones to collapse—was prevented by prior treatment with GDNF.^[^ ^87^ ^]^

### 3.4 Mechanisms of Regeneration in Adulthood

#### 3.4.1 CNS

Neural regeneration in the CNS was believed to be impossible due to several factors, including the expression of growth‐inhibiting proteins by oligodendrocytes, and the formation of scar tissue rich in chondroitin sulfate by astrocytes, at the site of injury.^[^ ^88^ ^]^ However, recent evidence has suggested that neuronal regrowth might be possible. Neural Stem Cells (NSC) located in the hippocampus and the lining of ventricles were found to generate both neurons and glia.^[^ ^89^ ^]^ Indeed, the ventricular Subventricular Zone (SVZ) is the largest germinal zone in the adult brains.^[^ ^90^ ^]^ Ventricular SVZ‐derived neurons have been shown to migrate to the olfactory bulb under physiological conditions, and toward injured areas when those are present.^[^ ^90^ ^]^ Glial cells were even shown to contribute to axonal recovery. In cases of genetic disorders causing axonal degeneration, astrocytes were found to surround and form a dense barrier protecting dystrophic axons.^[^ ^91^ ^]^ Stromal cells then formed fibrous connective tissue and deposited dense collagen as well as chondroitin sulphate proteoglycans, and oligodendrocyte progenitor cells proliferated.^[^ ^91^ ^]^ The role of microglia in axonal recovery seems to involve their differentiation into M1, which are pro‐inflammatory and neurotoxic, or M2 microglia, anti‐inflammatory and neuroprotective. Interleukin‐4 (IL‐4) and hemopexin are thought to modulate microglial differentiation. Several cytokines were shown to promote both phenotypes, and the complex mechanisms through which microglia differentiate and intervene are not fully understood.^[^ ^91^ ^]^

Several molecules have been shown to play critical roles in central nerve regeneration. TGF‐*β* is important for axonal recovery, following an injury. Fibroblasts secrete the signaling molecule, which attracts Schwann cells and causes them to migrate to the nerve stump, where they guide its regrowth.^[^ ^32^ ^]^ Following spinal cord injury, BMP was shown to be negatively involved in axonal regrowth in mice. Decreased BMP levels through genetic mutations and BMP antagonists resulted in an improved functional recovery. BMP2 and BMP4 increased the presence of oligodendrocytes and astrocytes at the site of injury, believed to hinder recovering axons.^[^ ^32^ ^]^ Wnt and its canonical pathway were activated following spinal cord and optic nerve injuries, and are believed to promote functional recovery of injured axons.^[^ ^32^ ^]^ Despite the complete mechanism still being unclear, its study could lead to treatments for nervous injuries.^[^ ^32^ ^]^ JNK was found to trigger neuronal apoptosis, and both axonal degeneration and regeneration.^[^ ^78^ ^]^ The JNK pathway has been shown to trigger neuronal degeneration in several neurological disorders, including ischaemia, epilepsy, or Alzheimer's disease.^[^ ^78^ ^]^ However, some studies found JNK to be necessary for neurite elongation during regeneration, and loss of JNK2 or JNK3 resulted in delayed neuritogenesis both in vitro and in vivo.^[^ ^78^ ^]^ NT‐3 attracted recovering adult rat corticospinal fibers, and induced regeneration.^[^ ^32^ ^]^ Increased BDNF levels in the site of spinal cord injury, in rats, resulted in improved locomotor recovery.^[^ ^32^ ^]^ However, contradicting results were found, as some studies did not observe any improvements.^[^ ^32^ ^]^ Differences in injury locations, between studies, were suggested to cause the contradictory evidence. BDNF would therefore have different effects, depending on the lesion site.^[^ ^32^ ^]^

#### 3.4.2 PNS

Injuries to the PNS are relatively frequent but often irreversible. Regeneration is slow and incomplete, rarely resulting in a complete functional recovery.^[^ ^68^, ^92^, ^93^ ^]^ Large injuries, or those that section the neuron close to its body, will result in neuronal death.^[^ ^93^ ^]^ However, following distant injuries, peripheral neurons can regenerate.^[^ ^93^ ^]^

Following a lesion, a rupture in the cell membrane triggers membrane depolarization, and a rise in intracellular Ca^2+^ concentrations.^[^ ^94^ ^]^ On both ends of the injury—proximal and distal—the membrane seals, the cytoskeleton is remodeled, and slow axonal retraction is observed.^[^ ^94^ ^]^ Then, the processes carried out on the proximal and distal sides differ. Regeneration of injured axons usually takes place on the proximal segment, which forms a growth cone, and develops in a similar fashion to neurons in prenatal development.^[^ ^94^ ^]^ The distal side degenerates.^[^ ^94^ ^]^

Degenerating axonal stumps can undergo either Wallerian degeneration or Axonal Degeneration (AxD), also known as Wallerian‐like degeneration.^[^ ^68^ ^]^ Wallerian degeneration refers to the decay of distal parts of an injured axon, while AxD comprises all forms of irreversible axon injury.^[^ ^68^ ^]^ Wallerian degeneration starts with the formation of axonal and myelin debris, which are phagocytosed through the activation of resident macrophages.^[^ ^93^ ^]^ Surrounding Schwann cells release chemoattractant proteins, which lead circulating monocytes to the site of injury.^[^ ^93^ ^]^ Increased mRNA translation can be observed in the injured neuron, which results in signaling molecules being guided to the neuron's body, informing of the damage.^[^ ^93^ ^]^ AxD includes axonal fragmentation, neurofilament scaffold disintegration, proliferation and activation of Schwann cells, dissolution then clearance of the myelin sheath and axon debris, and recruitment of inflammatory cells, including macrophages.^[^ ^68^ ^]^ Schwann cells form bands of Büngner, together with remaining connective tissue: these tubular structures guide sprouting axons.^[^ ^68^ ^]^ Interestingly, the degenerative process of the distal side seems to affect the regeneration taking place at the other end,^[^ ^94^ ^]^ and can even be favorable for the regeneration of the surviving end.^[^ ^92^ ^]^

VEGF was shown to play a role in peripheral nerve degeneration and regeneration. Muratori et al. studied VEGF family molecules in four different settings in rats: median nerve crush injury and median nerve transaction, followed or not by end‐to‐end surgical repair. VEGFR1, VEGFR2, VEGFR3, NRP1, and NRP2 were all modulated under degenerating and regenerating conditions.^[^ ^95^ ^]^ Immunohistochemical evidence suggested an autocrine VEGF/VEGFR2 pathway localized on Schwann cells.^[^ ^95^ ^]^ Activated macrophages at the site of injury secrete Interleukin‐1 (IL‐1), which induces the secretion of NGF by Schwann cells.^[^ ^93^ ^]^ TGF‐*β*, also released by macrophages, enhances Schwann cells’ mitotic activity, which reaches a peak 3 to 4 days after injury.^[^ ^93^ ^]^ BDNF and GDNF were also shown to be produced following neuronal injuries, and to promote recovery.^[^ ^93^ ^]^ Finally, neurotrophic factors, such as semaphorins, ephrins, and netrins, are believed to increase the chances of cell survival, and promote the secretion of previously mentioned growth factors.^[^ ^93^ ^]^ JNK, which has been implicated in complex and dual mechanisms during nerve degeneration and regeneration.^[^ ^96^ ^]^

A series of signaling molecules have been tested as potential treatments for peripheral nervous injury, but big enough sample sizes and clinical studies are still lacking. In mice, FGF2 overexpression resulted in Schwann cell proliferation and enhanced myelination, and doubled the number of regenerating axons.^[^ ^32^ ^]^ TGF‐*β* and Ephrins are believed to work together in directing injured axons.^[^ ^32^ ^]^ BMP2 and BMP7 respectively resulted in improved regeneration of facial nerves and the spinal cord in rats.^[^ ^68^ ^]^ NGF treatment was shown to support neurite outgrowth.^[^ ^68^ ^]^ NT‐3 and NT‐5 promoted the functional reinnervation of skeletal muscle, while NT‐4 and NT‐5 prevented cell death of embryonic rat spinal cord motor neurons in vitro, and NT‐4 increased axonal diameter and number, myelin thickness, and function after recovery.^[^ ^68^ ^]^ Treatment with BDNF increased recovery after spinal cord injury, while GDNF prevented retrograde motor neuron loss and atrophy in injured neonatal facial nerves.^[^ ^68^ ^]^

Other studied molecules include ciliary neurotrophic factor (CNTF), leukemia inhibitory factor (LIF), oncostatin M (OSM), hepatocyte growth factor (HGF), and artemin (ARTN), which all showed positive results, following an injury to peripheral nerves. CNTF, originally produced by parasympathetic cholinergic neurons, prevents axonal degeneration. Neonatal rats exposed to recombinant human CNTF had higher numbers of re‐growing axons. LIF was shown to support developing sensory neurons. Following an injury, it could enhance muscle contraction, conduction velocity, myelinated fiber number as well as their diameter. OSM is a neuroprotective cytokine of the Interleukin family, which attenuates neuronal death. Its effects were tested in mice whose paws were exposed to intense heat: subcutaneous injection of OSM resulted in reduced axonal withdrawal, counteracting the heat's damaging effects. HGF interacts with c‐Met Receptor Tyrosine Kinase. Rats whose nerves were crushed received intramuscularly injected HGF, which resulted in improved nerve function and structure. Finally, ARTN binds to GDNF receptor GFR*α*3, and acts through the PI3K‐AKT signaling pathway. Its levels are naturally increased following a nervous injury, and it was shown to enhance motor and sensory axonal recovery in rats.^[^ ^68^ ^]^

An extensive review by Bota et al. on drugs tested in the context of recovery from peripheral nerve injury, concluded that an outstanding amount of substances have been suggested and tested, yet none is currently used to accelerate peripheral nerve regeneration, due to small sample sizes and a lack of clinical studies.^[^ ^97^ ^]^

### 3.5 Summary

**Figure 4:**
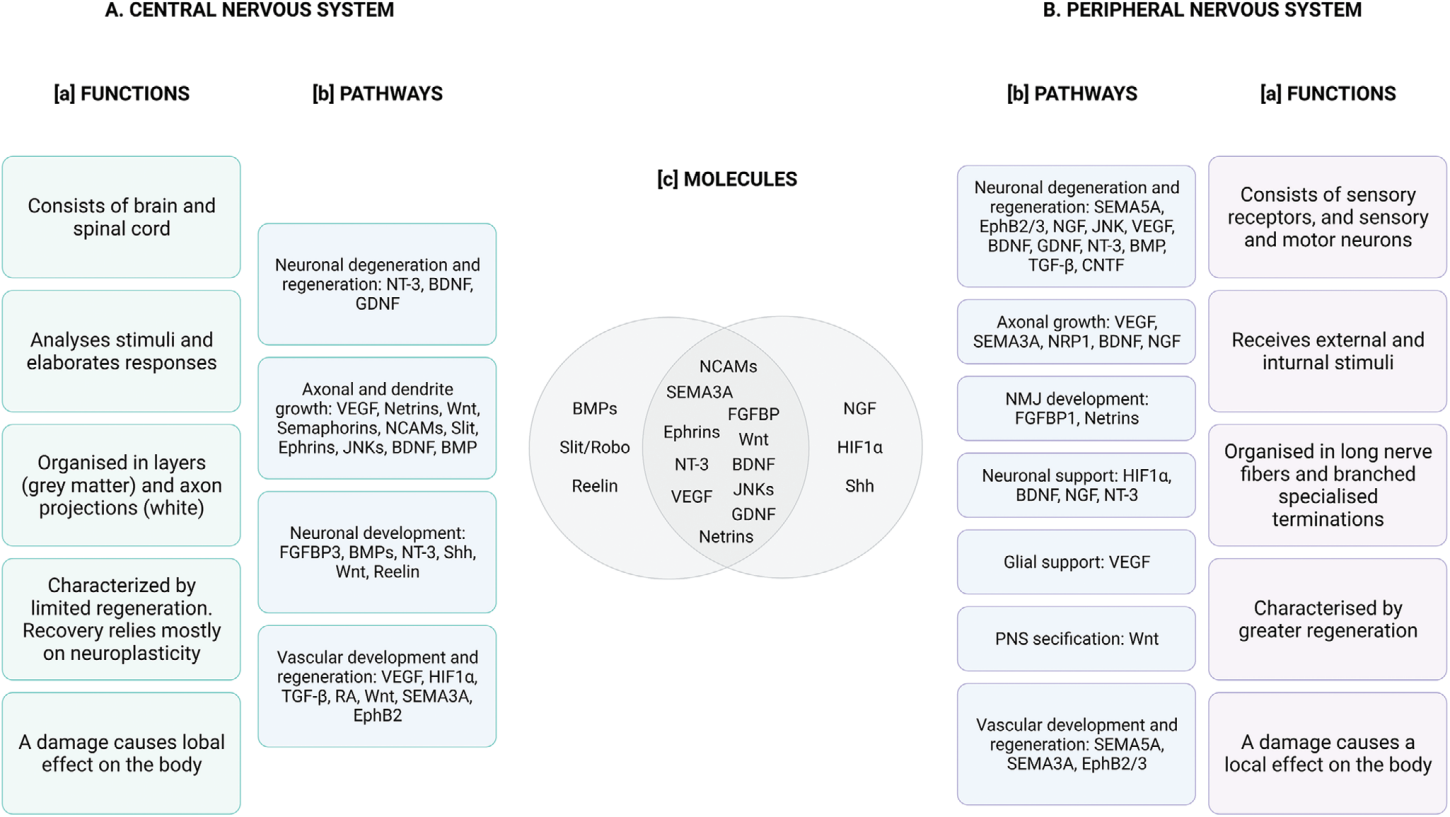
Parallelisms between central and peripheral nervous systems. The nervous system is divided into the A) CNS and B) PNS. The CNS consists of the brain and spinal cord, while the PNS is made of sensory and motor neurons in the rest of the body. Both systems are defined by their a) functional, b) signaling, and c) molecular differences. Regeneration is usually more successful in the PNS, while the CNS mainly relies on neuroplasticity to correct damage.

The nervous systems begins its development with the formation of the notochord and the primitive streak. The subsequent folding of the ectoderm forms the neural tube and crest, which later give rise to the CNS and PNS (Figure 1).^[^ ^2^, ^3^ ^]^ Within the nervous systems, neurons migrate to their destination using different methods. In areas of the CNS organized in layers, young neurons are guided by radial glial cells, while areas organized in nuclei do not rely on glia.^[^ ^2^ ^]^ In the PNS, the extracellular matrix and embryonic tissue guide neurons via surface molecules.^[^ ^2^ ^]^ Across the nervous system, growing neurons develop a common feature: growth cones. Although their function is comparable to that of tip cells—guiding developing neurons by sensing cues in the environment—they are an integral part of neurons instead of being separate cells (Figure 2).^[^ ^5^, ^32^, ^33^ ^]^ Growth cones are guided by two main types of molecular cues: chemoattractants and chemorepulsants, which they are respectively attracted to and repelled by. Chemoattractive cues include NT‐3, NGF, BDNF, and GDNF.^[^ ^32^, ^68^, ^75^ ^]^ Chemorepulsive cues include Slit, Shh, and SEMA3A.^[^ ^32^, ^33^, ^34^ ^]^ A few molecules’ effects on growth cones are still unclear, such as BMP7.^[^ ^32^ ^]^ On top of growth cone guidance, neuronal development relies on complex processes such as synapse formation and midline crossing. Synapse formation involves several processes, which range from the maturation of presynaptic regions to selective synapse elimination. Among others, FGFs, SEMAs, BDNF, and NGF are believed to be at play (Figure 3).^[^ ^5^, ^34^, ^62^, ^64^, ^68^ ^]^ Midline crossing is the process through which commissural axons in the CNS travel from one hemisphere to the other, and is thought to involve Slit and Robo, as well as Netrins, JNKs, and Wnt.^[^ ^32^, ^33^, ^70^, ^71^, ^72^, ^78^ ^]^ Finally, neural regeneration, especially in the CNS, is an extremely interesting field of research which is currently under investigation. Indeed, neurogenesis in the CNS was believed to stop after development (**Figure** **4**).^[^ ^88^ ^]^ However, recent evidence has suggested that regrowth and recovery in adulthood might be possible.^[^ ^89^, ^90^, ^91^ ^]^ TGF‐*β*, Wnt, JNKs, NT‐3, and BDNF have already been shown to play a role in the regenerating CNS,^[^ ^32^, ^78^ ^]^ while BMP supposedly hinders it.^[^ ^32^ ^]^ Regarding regeneration in the PNS, the mechanisms are different to the CNS and better understood. Although complete functional recovery is rare,^[^ ^68^, ^92^, ^93^ ^]^ the proximal segment of lesioned axons can form growth cones and develop in a similar fashion to neurons in prenatal development.^[^ ^94^ ^]^ VEGF, NRPs, NGF, TGF‐*β*, BDNF, GDNF, JNKs, as well as SEMAs, Ephrins, and Netrins are believed to be involved in PNS regeneration.^[^ ^93^, ^95^, ^96^ ^]^ Overall, axonal regeneration is still under research, and a clearer understanding of developmental processes could bring light onto regenerative ones, and recent findings regarding regeneration in the CNS are especially promising.

## 4 Neurovascular Unit

### 4.1 Research History

**Figure 5:**
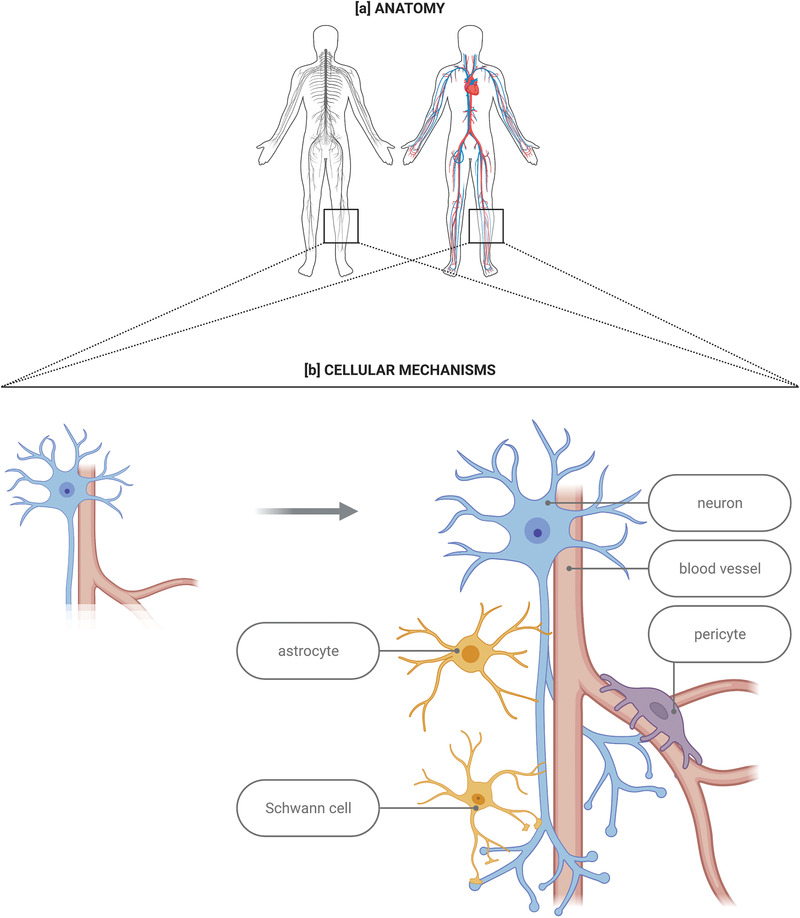
Parallel development of nerves and blood vessels. a) Similarities in the anatomical structures of the human nervous and vascular systems include their branching shape and coverage of the entire body. b) Nerves and blood vessels often develop side by side, and influence each other's growth. Vascular cells, neurons, astrocytes, oligodendrocytes or Schwann cells, and pericytes are part of the NVU and probably interact with one another to coordinate development.

In 2001, the concept of neurovascular unit (NVU) was formalized by the National Institute of Neurological Disorders and Stroke,^[^ ^98^ ^]^ which is part of the National Institutes of Health of the Unites Stated of America. The NVU establishes the relationship between the brain and its blood circulation,^[^ ^98^ ^]^ and comprises cells relevant in the crosstalk between the vascular and neural systems: namely neurons, glia, pericytes, extracellular matrix, circulating blood cells, and blood vessel walls and their surrounding smooth muscle cells (**Figure** **5**).^[^ ^99^ ^]^

Since the characterization of the NVU, growing interest has been shown by the scientific community in the mechanisms through which the vascular and neural systems interact, as can be attested by the increasing amount of publications released every year.

Recently, these interactions have been approached from different perspectives, and several new concepts arose defining those levels of study. Neurovascular Coupling is the process by which support cells regulate blood flow in response to neural activity,^[^ ^100^, ^101^ ^]^ while the Neurovascular Niche is a microenvironment made of neural progenitor cells and their interactions with the vasculature; it is believed to regulate adult neurogenesis.^[^ ^6^, ^51^, ^102^ ^]^

### 4.2 Mechanisms of Development at the Cellular Level

#### 4.2.1 Vascular Influence on the Neural System

It is clear that neurogenesis somehow depends on components of the vascular system. In specific areas of the nervous system, such as the neurogenic subgranular zone of the hippocampus or the SVZ, vascular niches are present and NSCs proliferate in close contact with dividing capillaries.^[^ ^103^ ^]^ However, little is known on the exact implications and effects of the vascular system on neurogenesis. Perturbations in blood vessel growth have resulted in flawed positioning of Neural Progenitor Cells (NPCs), often found in non‐neurogenic areas. Yet, these results are not always consistent. NPCs in dorsal regions, giving rise to excitatory neurons, do not seem to require blood vessels, contrarily to NPCs from the ventral telencephalon which give rise to inhibitory neurons.^[^ ^6^ ^]^

Blood vessel formation is known to modulate the balance between NPC proliferation and differentiation. For example, hypoxic neurogenic niches enhance NPC proliferation, while an increase in blood supply through angiogenesis induces NPC differentiation. HIF1*α* mediates this process.^[^ ^6^ ^]^

Vascular cells and blood vessels can guide the migration of both neurons and oligodendrocytes. Vascular‐mediated guidance of developing neurons appears mostly in the prenatal and infant brain, but is also present in the adult brain. For instance, neuroblasts from the posterior SVZ were shown to migrate to the anterior olfactory bulb, with the help of blood vessels.^[^ ^6^ ^]^ Blockade of VEGF signaling in the developing vasculature resulted in dysfunctional migration of GABAergic neurons.^[^ ^6^ ^]^ In some cases, astrocytes associate with blood vessels to orchestrate neuronal migration.^[^ ^6^ ^]^ Oligodendrocyte precursors, through Wnt‐dependent CXCR4 expression, were attracted to CXCL12‐expressing endothelial cells.^[^ ^6^ ^]^ Once they have reached their target, Wnt signaling is decreased, resulting in detachment from the vasculature, and oligodendrocyte maturation.^[^ ^6^ ^]^ A study on rodent brains showed that abrupt changes on baseline vascular tone can affect the firing activity of pyramidal neurons.^[^ ^104^ ^]^ In the PNS, a study performed on mouse limb skin revealed that neurons favored arterial vessels over veins, to determine the direction of their development.^[^ ^105^ ^]^

#### 4.2.2 Neural Influence on the Vascular System

The intracerebral vasculature follows a specific pattern while developing. It begins by growing in a converging manner toward the ventricles, which it surrounds, before migrating in the opposite direction, toward the pial surface.^[^ ^106^ ^]^ Since the cerebral microvasculature has to meet the brain's high oxygen demands, its development has been hypothesized to depend on the neural system. Neurons and glial cells are believed to attract developing endothelial cells and regulate their migration.^[^ ^6^ ^]^ However, the mechanisms through which cells of the nervous system influence vascularisation remain to be elucidated. Brain activity has been tested as a potential regulator of vascular development and was shown to affect cerebral angiogenesis, but findings are contradictory. Lacoste et al. reported that an increase in sensory neuronal activity resulted in enhanced endothelial cell proliferation, vascular density, and branching in mouse pups, while sensory deprivation had the opposite effects.^[^ ^107^ ^]^ However, Whiteus et al. found no effects of reduced sensory input on vascular development.^[^ ^108^ ^]^ Excessive sensory stimulation has led to reduced endothelial cell proliferation and vessel sprouting in multiple studies.^[^ ^6^, ^109^ ^]^

Considerably less is known about the molecular processes taking place in the PNS. Although studies from over a decade ago have shown the potent pro‐angiogenic effects of NPCs in co‐cultures both in vitro and in vivo,^[^ ^110^ ^]^ the molecular mechanisms behind such interactions are unclear. Glial cells within the PNS are believed to promote endothelial regeneration. Wang et al. performed a study on rats, examining the processes taking place after sciatic nerve transection, and found 16 genes that participated both in vascular and nervous regeneration, including SEMA5A, EphB2, and EphB3.^[^ ^111^ ^]^ Ramos et al. assessed the cross‐talk between the vascular and neural systems, with the use of human umbilical vein endothelial cells and rat Schwan cells.^[^ ^112^ ^]^ Their results showed a considerable increase in area covered by the developing vasculature, when co‐cultured with Schwann cells, as opposed to single endothelial cell cultures.^[^ ^112^ ^]^ VEGF and FGFR3 were both upregulated in those co‐cultures.^[^ ^112^ ^]^

#### 4.2.3 Formation of the Blood‐Brain Barrier (BBB)

The BBB is a complex structure that separates the CNS from its vasculature. It consists of endothelial cells, pericytes, and astrocytes which are in contact with neural tissue.^[^ ^113^ ^]^ The formation of the BBB and vascularisation of the CNS start with the formation of a perineural vascular plexus, by angioblasts entering the head region.^[^ ^113^ ^]^ Growing vessels then elongate via angiogenesis, radially invading neural tissue, and form a network of capillaries by anastomosing with one another.^[^ ^113^, ^114^ ^]^ Molecules such as VEGF, Wnt, and the Hh pathway are believed to be involved in BBB formation.^[^ ^114^ ^]^ G‐Coupled Protein Receptor 124 (GPR124), a G‐protein coupled receptor, has also been shown to be essential for cerebral vascularisation, as mice knockouts resulted in an inability of the vascular sprouts to invade the nervous tissue, resulting in the animals’ death.^[^ ^114^ ^]^ Endothelial cells also undergo phenotypic changes to match the needs of the BBB. Among others, they express Glut‐1, a glucose transporter whose presence is one of the earliest BBB markers, and P‐glycoprotein, which is required for BBB differentiation.^[^ ^113^ ^]^ On top of that, the BBB has to develop its permeability. This is believed to take place gradually, as angiogenesis occurs, rather than simultaneously taking place across the entire BBB.^[^ ^113^ ^]^ Interestingly, the differentiation of endothelial cells toward BBB characteristics has been shown to be a result of their proximity to neural cells, rather than a pre‐determined preference.^[^ ^113^ ^]^ This further emphasizes the importance of neurovascular crosstalk in development.

### 4.3 Mechanisms of Development at the Molecular Level

#### 4.3.1 VEGF

VEGF modulates neural control of vascular development (Figure 3). Astrocyte and neuronal‐derived VEGF were both shown to affect developing blood vessels by modulating the directions taken by their tip cells.^[^ ^6^ ^]^ Amacrine and horizontal neurons in the retina were shown to express VEGF in an HIF1*α*‐dependent manner, in order to control blood vessel sprouting and branching.^[^ ^6^ ^]^ In addition, peripheral neurons are believed to induce arterial differentiation through VEGF, which regulates EphrinB2 expression.^[^ ^105^ ^]^ VEGF secretion from peripheral neurons significantly decreased in the absence of Schwann cells, in vitro.^[^ ^105^ ^]^

Neural tube vascularization is highly VEGF‐dependent. The formation of a Perineural Vascular Plexus (PNVP) surrounding the neural tube, from which vessel sprouts arise, was hindered by VEGF inhibitors in vitro.^[^ ^51^ ^]^ When presomitic mesoderm explants from VEGFR2‐deficient embryos were cocultured with wild‐type neural tubes, PNVP formation was inhibited.^[^ ^51^ ^]^ NSCs, NPCs,^[^ ^31^ ^]^ and glial cells^[^ ^6^ ^]^ are thought to secrete VEGF to regulate the formation of PNVP. Shh has been shown to regulate VEGF expression in the neural tube, ensuring its timely and highly specific action.^[^ ^115^ ^]^

VEGF plays a role in vascular control of neural development as well (Figure 3). Endothelial cell‐derived VEGF was shown to promote proliferation, differentiation, and migration of NSCs and NPCs, oligodendrocyte precursors, and neurons.^[^ ^6^ ^]^ VEGF has been studied in the context of vascular sympathetic innervation, where VEGF receptors, present in sympathetic nerve fibers innervating arteries, respond to VEGF produced by vascular cells.^[^ ^116^ ^]^ Additionally, VEGF produced by vascular smooth muscle cells in sympathetic neurovascular cocultures, through VEGFR1, inhibited the action of SEMA3A, and the growth cone collapse it is responsible for.^[^ ^116^ ^]^

VEGF is thought to regulate blood vessel patterning in the spinal cord, through interactions with endothelial cell‐derived Soluble Fms‐Like Tyrosine Kinase‐1 (sFlt1).^[^ ^6^ ^]^ Astrocyte‐Derived Glial Fibrillary Acidic Protein (GFAP) is also believed to affect VEGF signaling in a neurovascular context.^[^ ^29^ ^]^ Slit2 has shown inconsistent effects on VEGF signaling. It sometimes induced VEGF internalization, therefore inhibiting its effects, while it has also resulted in increased endothelial cell motility when co‐induced with VEGF.^[^ ^6^ ^]^

#### 4.3.2 Delta/Notch

Little is known on the Notch signaling pathway, in the context of neurovascular development. Jagged1, one of the ligands of the Delta/Notch signaling pathway, was secreted by endothelial cells and critical for maintaining NSCs from the SVZ in a quiescent state, by inhabiting their differentiation together with EphrinB2.^[^ ^6^, ^117^ ^]^ Deletion of either Jagged1 or EphrinB2 in stem cells resulted in premature activation and neuronal differentiation.^[^ ^117^ ^]^ Another study examined the role of Notch receptors—especially Notch1, Notch3, and Notch4—in relation to neurovascular development, in the context of pancreatic cancer. They were shown to enhance neurovascular development in tumors.^[^ ^118^ ^]^

#### 4.3.3 Semaphorins (SEMA)/Plexins

Semaphorins and Plexins have been shortly mentioned in literature, in relation to neurovascular development. SEMA3E, produced by retinal ganglion cells, was shown to signal to endothelial cells through PlexinD1.^[^ ^119^ ^]^ Ultimately, this pathway prevented the developing vasculature from growing in the wrong direction in the retina.^[^ ^119^ ^]^ Interestingly, it appeared that SEMA3E concentrations were homogenous across the retina. An endothelial cell's responsiveness therefore depended on its expression of PlexinD1, whose levels were regulated by VEGF ligands.^[^ ^120^ ^]^ VEGF could play a dual role, as a chemoattractant itself and by enhancing chemorepulsive cues through PlexinD1.^[^ ^120^ ^]^ In the PNS, SEMA3A mutants showed disrupted vascular patterns, which aligned with the abnormally remodeled nerves.^[^ ^105^ ^]^

#### 4.3.4 TGF‐*β*

TGF‐*β* plays a role in neural control of vascular development, especially in the CNS. There, neural progenitors express *β*8 integrin, activating extracellular matrix‐bound latent TGF‐*β*, which signals to developing endothelial cells. NRP1 expressed by endothelial cells then forms intercellular protein complexes, with *β*8 integrin found on NPCs. Deactivation of TGF‐*β*, *β*8 integrin, or NRP1 all individually led to angiogenesis defects in the brain. In the retina, TGF*β*R2 was involved in endothelial cell migration during vascularization. Furthermore, the receptor GPR124 is believed to play a role in TGF‐*β*‐dependent cerebral angiogenesis. The germinal matrix, a primordial brain structure that gives rise to the striatum, was found to rely on a unique TGF‐*β*‐dependent pathway. Neural progenitors in the germinal matrix regulate their *β*8 integrin expression through Sphingosin‐1‐Phosphatase. It is still unknown whether TGF‐*β* signaling could be area‐specific and contribute to brain structure differentiation. Regarding the vascular control of neural development, pericyte‐derived TGF‐*β* was shown to repel oligodendrocyte precursors from the ventral forebrain, favoring their dorsal migration toward the cortex.^[^ ^6^ ^]^

#### 4.3.5 Retinoic Acid (RA)

RA has been studied in relation to neurovascular development, especially in neural control of vascular development. Mizee et al., in their study performed on human post‐mortem foetal brain tissue, concluded that radial glial cells express retinaldehyde dehydrogenase, an enzyme responsible for RA synthesis, while the developing brain vasculature expresses RAR*β*.^[^ ^121^ ^]^ Pharmacological inhibition of RAR in mice resulted in enhanced BBB leakage and the presence of serum proteins in the developing brain, suggesting a significant effect of RA on the development of the BBB.^[^ ^121^ ^]^ Additionally, RA was shown to regulate VEGF and Wnt signaling pathways during vascular development in the CNS. It could stimulate VEGFA derived from neural stem and progenitor cells, which was previously said to be involved in the development of PNVP.^[^ ^31^ ^]^ Mice locally lacking RA developed a flawed cerebrovasculature, especially in the PNVP overlaying the cerebral cortex.^[^ ^31^ ^]^ RA inhibits Wnt signaling inhibitors, thus enhancing Wnt activity.^[^ ^122^ ^]^ Mice globally lacking RA showed important cerebrovascular defects, which correlated with decreased levels of Wnt signaling.^[^ ^122^ ^]^

#### 4.3.6 Erythropoietin‐Producing‐Hepatocellular Carcinoma Receptors (EphR)/Eph Receptor‐Interacting Signals (Ephrins)

Ephrins and their receptors have been studied in relation to the BBB and the development of the cerebral vasculature. Their level of expression was related to BBB integrity.^[^ ^123^ ^]^ Increased levels of EphrinA1, for instance, led to increased permeability.^[^ ^123^ ^]^ Ephrins also mediate the crosstalk between the developing vasculature and glial cells. Endothelial cells expressing EphA4 attracted radial glial cells, which support early angiogenesis through the formation of a physical scaffold.^[^ ^123^ ^]^ In mice, EphrinA5 expressed on astrocytes, and EphA4 found on endothelial cells, were shown to interact and be especially important in the development of the vasculature of the hippocampus.^[^ ^123^ ^]^ Additionally, Ephrins were shown to play a role in Schwann cell invasion of the CNS, in certain pathophysiological conditions, both in vitro and in vivo.^[^ ^124^ ^]^ EphrinB3, especially, is believed to be present in healthy myelin and repel Schwann cells, which could explain their attraction to CNS axons in cases of demyelination.^[^ ^124^ ^]^ Finally, EphrinB2 expressed by endothelial cells inhibited the differentiation of neonatal neural stem cells in the SVZ, thus being critical for their maintenance.^[^ ^6^ ^]^

#### 4.3.7 Wingless/Int‐1 (Wnt)

Wnt signaling is necessary for correct vascularization of the CNS. Indeed, alterations in both Wnt7a or Wnt7b signaling in endothelial cells resulted in decreased blood vessel formation, vascular malformations, and BBB dysfunction.^[^ ^6^ ^]^ Endothelial cells in the CNS express receptor Frizzled6, while Wnt7a and Wnt7b are expressed by neurons, NSCs, and NPCs.^[^ ^31^ ^]^ GPR124 and its cofactor Reversion‐Inducing Cysteine‐Rich Protein With Kazal Motifs (RECK) are believed to mediate this pathway as coactivators.^[^ ^6^, ^31^ ^]^ In the retina, Wnt was shown to act through a different set of molecules, such as Norrin, derived from Müller glial cells and bound to Frizzled4 with coreceptor Lrp5 and coactivator Tspan12. This pathway was present in the vascularization of the CNS as well, complementarily to the GPR124‐dependent mechanism.^[^ ^6^ ^]^

#### 4.3.8 BDNF

BDNF is expressed and secreted by vascular endothelial cells in the brain, and acts both as a migration cue and a neuroprotective signal.^[^ ^102^, ^125^, ^126^ ^]^ Human endothelial cell‐derived BDNF was shown to stimulate axonal growth in chicken and rat dorsal root ganglia.^[^ ^126^ ^]^ BDNF levels in neurons were regulated by Cyclic Adenosine Monophosphate (cAMP),^[^ ^125^ ^]^ and BDNF expressed by endothelial cells was shown to act upon neuronal migration through p75NTR, a receptor expressed on neuroblasts.^[^ ^127^ ^]^ GABA was released from neuroblasts, and induced TrkB receptor invagination, thus inhibiting BDNF signaling.^[^ ^127^ ^]^

#### 4.3.9 GABA

GABA has been defined as the main excitatory neurotransmitter present in the developing brain and modulates cell proliferation, neuroblast migration, and dendritic maturation through epigenetic mechanisms.^[^ ^128^ ^]^ It can also be produced by endothelial cells, and regulates the tangential migration of GABAergic neurons.^[^ ^6^ ^]^ GABA has been related to the development of a functional cortical structure: endothelial cells across different brain areas express different GABA receptors, and guide developing neurons to their destination. Pial endothelial cells, found in the innermost layer of the meninges, express GABRA2, which guides the migration of superficial neurons, whereas periventricular endothelial cells express GABRB3, guiding inner deep neurons.^[^ ^6^ ^]^ Additionally, GABA is believed to enhance angiogenesis in the CNS. A study on the vasculature of the paraventricular nucleus of the hypothalamus—an area known for having a high density of blood vessels in the brain—concluded that a disruption of GABA receptors resulted in decreased vascular density.^[^ ^129^ ^]^ Distorted GABA levels during embryonic development have been suggested to be related to several psychiatric diseases, which have both vascular and neural components, such as schizophrenia, depression, or epilepsy.^[^ ^130^ ^]^ Interestingly, several drugs used in obstetrics modulate GABA receptors’ function, and further investigation of this phenomenon was encouraged.^[^ ^130^ ^]^

#### 4.3.10 Renin‐Angiotensin System (RAS)

RAS involves Angiotensinogen, which is cleaved by Renin to form Angiotensin I (AngI). Angiotensin‐Converting Enzyme (ACE) further converts AngI into AngII, which can bind to two receptors: Angiotensin Receptor 1 (AT1‐R) and AT2‐R. Another enzyme, ACE2, can turn AngI into Angiotensin 1–9.^[^ ^100^ ^]^ Neurons, glia, and blood vessels of the retina express AT1‐R, while AT2‐R is expressed by inner retinal neurons. Glial cells are thought to produce AngII, which is therefore a strong candidate for modulating communication between neurons, glia, and endothelial cells. AT1‐R and AT2‐R have respectively been related to retinal vasoconstriction and vasodilation, while AngII has a wide role in retinal homeostasis, including blood vessel constriction, regulation of glial cell function, and modulation of neuronal function. Recently, a special focus has been put on RAS‐dependent activation and regulation of microglia function. RAS‐antagonists reduced microglial activation, while AngII triggered rat microglia via ATR‐1.^[^ ^100^ ^]^

### 4.4 Mechanisms of Regeneration in Adulthood

#### 4.4.1 Mechanisms at the Cellular Level

Following a lesion in the CNS, such as in the case of stroke, new neurons arising from the V‐SVZ were found to rely on blood vessels for their migration toward the injured area, more than they did under physiological conditions.^[^ ^90^, ^131^ ^]^ Molecules that mediate the contact between neuroblasts and blood vessels include diffusible molecules and extracellular matrix proteins, such as type 4 collagen, laminin, or fibronectin.^[^ ^90^ ^]^

In the PNS, the two stumps of a fractured neuron can be joined through a structured defined as “the bridge,” which comprises macrophages, neutrophils, fibroblasts, and endothelial cells. A study on rats showed that the proportions in which the different components of the bridge are present vary, as an injury recovers. The amount of endothelial cells, for example, significantly increases between the second and third day following the injury, suggesting a vascularisation of the bridge. Macrophages, through their secretion of VEGFA, have been shown to induce the formation of a polarized endothelial scaffold, which directed Schwann cells across the bridge. Indeed, Schwann cells were shown to migrate along newly constructed blood vessels, both in vitro and in vivo.^[^ ^132^ ^]^

#### 4.4.2 Mechanisms at the Molecular Level

VEGF was shown to play a role in neurovascular regeneration. Inhibition of its signaling resulted in degradation of the BBB,^[^ ^133^ ^]^ while its activation was reported to induce the differentiation of NSCs into neuroblasts, in both humans and mice.^[^ ^134^ ^]^ In embryonic mouse limb skin, VEGF derived from peripheral nerves was shown to promote arterial differentiation.^[^ ^135^ ^]^ Notch and Ang1 are important in cases of hypoxia.^[^ ^136^, ^137^ ^]^ Notch3 signals through Jagged1 and promotes the maturation of pericytes, which in turn secrete AngI. Binding of AngI to TIE2 promotes the survival of endothelial cells, strengthens cellular adhesions, and increases pericyte coverage.^[^ ^137^ ^]^ AngI was also found to enhance endothelial cell migration and tubulogenesis, defined as the formation of tubes during vascular development.^[^ ^136^ ^]^ Since TIE2 is also expressed on neurons, AngI is a strong candidate for simultaneously promoting neural and vascular regeneration. Hh signaling was shown to promote BBB integrity in both embryos and adults, by promoting the expression of tight and adherens junction proteins in endothelial cells and decreasing the barrier's permeability.^[^ ^42^ ^]^ Finally, BDNF produced by endothelial cells acts as a neurotrophic factor against ischemia. *β*1 integrin and Integrin‐Linked Kinase (ILK) signaling have been found to promote the production of BDNF.^[^ ^133^ ^]^

#### 4.4.3 Focus on Pericytes

Pericytes were previously mentioned in this review as being involved in adult regeneration of the vascular system, and as being part of the NVU. This fact alone is already an indicator of the promising roles they play in the interplay between both systems during regeneration. Additionally, pericytes exhibited plastic behavior in response to injury: the ablation of single pericytes resulted in the extension of neighboring pericytes to cover exposed endothelium.^[^ ^138^ ^]^ Furthermore, aside from supporting vascular function^[^ ^139^ ^]^ and acting as phagocytes,^[^ ^140^ ^]^ they are capable of differentiating into a vast array of cell types—including mesenchymal lineage, neurons, astrocytes, and oligodendrocytes—which have put them under the focus of current research.^[^ ^136^ ^]^ However, pericytes found across tissues seem to differentiate into distinct lineages, suggesting the presence of pre‐programmed preferences,^[^ ^37^ ^]^ and there is increasing evidence that pericytes residing in the CNS, and those found in the PNS, have molecular and functional differences.^[^ ^141^ ^]^ Some molecules, referred to as “lineage drivers,” have been linked to specific differentiation fates. For example, myocardin drives differentiation into smooth muscle cells, while transcription factor Runx2—which is upregulated by BMP—was related to osteogenic and chondrogenic differentiation.^[^ ^37^ ^]^ Peroxisome proliferator activated receptor gamma (PPAR*γ*) is a transcription factor that regulates differentiation into adipocytes.^[^ ^37^ ^]^ Some pericytes exhibit molecular markers, which also differ between and within lineages. For example, both Nerve/Glial Antigen 2 (NG2)‐positive and negative pericytes can be found in the skin.^[^ ^142^ ^]^ Capillary pericytes are usually *α* Smooth Muscle Actin (*α*SMA)‐negative, while venular pericytes express *α*SMA.^[^ ^143^ ^]^ Pericytes of different origins and expressing different markers differ in their functions: a pericyte subtype could promote regeneration while another could hinder it.^[^ ^144^ ^]^

Pericytes were studied in relation to the BBB. They could reduce its integrity through VEGF and Matrix Metallopeptidases, and increase it via AngI and TGF‐*β*. The latter supports tight junctions between endothelial cells, upregulates the release of ACE2 and extracellular matrix proteins—including fibronectin and collagen type IV—, and collaborates with BMP and Notch to enhance N‐cadherin expression between pericytes and endothelial cells. In fact, Notch signaling was also shown to reduce both pericyte migration and angiogenic function, through DLL4 and Notch3.^[^ ^136^ ^]^

Pericytes were shown to modulate recovery in a series of injuries and disorders. In the case of ischaemic injury, several growth factors induced pericyte activity, through PDGFRB and its bFGF‐dependent signaling,^[^ ^136^ ^]^ which leads to pericyte relaxation, allowing blood vessels to dilate and increase microcirculation nearby an injured area of the brain.^[^ ^139^ ^]^ Pericyte deficiency led to increased leakage of the BBB, while absence of PDGFRB signaling resulted in micro‐vessel aneurysms and brain haemorrhage, and its upregulation protected the BBB.^[^ ^136^ ^]^ Following a stroke, pericytes increased their expression of NT‐3, BDNF, GDNF, and NGF, which have neuroprotective effects.^[^ ^136^, ^139^ ^]^ In hypoxic conditions, HIF1*α* stimulates VEGF, resulting in enhanced pericyte proliferation and angiogenesis, while overactive angiogenesis can be detrimental to the BBB.^[^ ^136^ ^]^ Pericytes were found to have beneficial effects in brains suffering from Alzheimer's Disease and multiple sclerosis, respectively through enhanced brain microcirculation and phagocytosis of amyloid *β* deposits, and improved re‐myelination.^[^ ^136^ ^]^

### 4.5 Summary

Research on the NVU is much more recent than that of the vascular and nervous systems, and our understanding of its development and regeneration is therefore more scarce. The NVU is made of vascular and neural cells, and is defined by their interactions.^[^ ^98^ ^]^ During development, the vascular system influences the nervous system, and vice‐versa. For example, blood vessel formation is known to modulate the balance between NPC proliferation and differentiation.^[^ ^6^ ^]^ Vascular cells and vessels are also believed to guide the migration of neurons and oligodendrocytes.^[^ ^6^ ^]^ A series of signaling molecules have been shown to be involved in vascular control of neural development. Endothelial‐derived VEGF promotes proliferation, differentiation, and migration of neurons,^[^ ^6^ ^]^ while Notch maintains NSCs in their quiescent state,^[^ ^117^ ^]^ and BDNF acts as a migration cue.^[^ ^102^, ^125^, ^126^ ^]^ GABA, which is also expressed by vascular cells, is essential to the formation of a functional cortical structure.^[^ ^6^ ^]^ Endothelial cells across different areas express different GABA receptors, which guide neurons to their correct destinations.^[^ ^6^ ^]^ Simultaneously, the nervous system also influences vascular development. For example, the cerebral vasculature has been suggested to develop according to brain activity and neuronal patterns, in order to meet the brain's high oxygen demand.^[^ ^6^, ^107^ ^]^ A series of molecules have been identified which play important roles in nervous control of endothelial development. Neural cells expressing VEGF, SEMA3E, and Wnt can guide tip cells, affect vascular differentiation and promote endothelial regeneration.^[^ ^6^, ^105^, ^112^, ^119^, ^120^ ^]^ In the CNS, TGF‐*β* is activated by neural progenitors and involved in cerebral vascularization.^[^ ^6^ ^]^ RA, ephrins, and Wnt are believed to be important in the formation of the BBB.^[^ ^6^, ^121^, ^123^ ^]^ With regards to regeneration in adulthood, the vascular and nervous systems have been shown to promote each other's recovery. For example, in the PNS, the formation of a “bridge,” which joins the two stumps of a fractured neuron, includes endothelial cells.^[^ ^132^ ^]^ At the molecular level, VEGF, AngI, Hh signaling, and BDNF have been shown to mediate crosstalk between neural and endothelial cells.^[^ ^42^, ^133^, ^134^, ^135^, ^136^, ^137^ ^]^ On top of the aforementioned mechanisms, pericytes appear to be of particular importance in the regenerating NVU. Pericytes are cells that can be found along endothelial walls. They act as phagocytes as well as being capable of differentiating into a vast array of cell types, including mesenchymal lineage, neurons, astrocytes, and oligodendrocytes.^[^ ^136^, ^139^, ^140^ ^]^ Pericytes have been involved in BBB regeneration through VEGF, AngI, and TGF‐*β*.^[^ ^136^ ^]^ They have also been shown to modulate recovery in ischaemia through PDGF‐B, and in cases of stroke via NT‐3, BDNF, GDNF, and NGF.^[^ ^136^, ^139^ ^]^ Overall, pericytes are extremely promising in the context of neurovascular regeneration and treatment for degenerative disorders.

## 5 Perspectives for Regenerative Medicine

### 5.1 Research History

Compared to other species, the complexity of the human body makes it extremely difficult to repair and regenerate. A first clinical solution to this problem was found at the beginning of the 50s with the first organ transplants. Along with the improvements of transplant medicine, cell biology advanced toward the first organotypic cell cultures, where different cell types were seeded together to study their interactions. This was the first step toward tissue engineering. Rapid advances were made and, in 1998, the first allogeneic engineered skin, the “Apligraph,” reached the market. On the other hand, improvements in genetics, with Dolly the sheep being cloned in 1997,^[^ ^145^ ^]^ and the discovery of the first stem cells^[^ ^146^ ^]^ marked the dawn of stem cell biology. Eventually, tissue engineering and stem cell biology were merged and together they shaped the field of regenerative medicine, which now includes methods aimed at regrowing, repairing or replacing damaged or diseased cells, tissues, or even organs.

Nowadays, the evolution of regenerative medicine is proceeding rapidly. In 2006, Induced Pluripotent Stem Cells (iPSCs) were derived from reprogrammed adult somatic cells. Today, stem cells can be used for regenerative and transplant medicine,^[^ ^147^, ^148^ ^]^ disease modeling and drug screening,^[^ ^149^ ^]^ and human developmental biology.^[^ ^150^ ^]^ Translational research brought regenerative medicine to the clinic: in 2017, iPSC‐derived retinal cells were transplanted in a woman suffering from advanced macular degeneration.^[^ ^151^ ^]^ Applications for the treatment of complex neurological disorders were also found, and the first clinical trial for Parkinson's disease using allogenic iPSCs was approved in 2018.^[^ ^152^ ^]^ Dedicated infrastructures were built such as the iPSC therapy center at Kyoto university in Japan, and significant financial investments have been made to support regenerative medicine from both public authorities and industrial partners.^[^ ^153^ ^]^ The improvements in manufacturing and engineering have significantly accelerated the evolution of regenerative medicine. 3D‐bioprinting finds application to design scaffolds and cell‐laden constructs that can be implanted by reconstruction surgery interventions.^[^ ^154^ ^]^ Organ‐on‐chip devices allow the study of biological processes in a straight‐controlled environment, and enable more reliable drug screening tests for pharmaceutical applications.^[^ ^155^ ^]^ However, despite having gained the ability to artificially generate a great variability of cell lines and tissue, the scientific community is still far from developing functional organs. For this purpose, the understanding of signaling pathways is essential. Signaling molecules are crucial for the development and regeneration of tissues and organs, but their pleiotropic and synergic properties make them extremely difficult to study. Therefore, for diagnostic and therapeutic purposes, great attention is now pointed toward unraveling the mechanisms of action of signaling pathways for regenerative medicine.

### 5.2 Signaling Molecules and Pharmaceutical Drugs in the CNS

When damaged, the adult vascular and neural systems rely on similar molecular mechanisms to regenerate. Despite the attempts to unveil such complex molecular interactions, therapeutical applications with neurovascular signaling molecules have been already under investigation as possible treatments for neurovascular disorders.

As a key player in both angiogenesis and vascular permeability, VEGF is one of the most studied neurovascular factors that find applications for treatment in both neurodegenerative and cerebrovascular disorders.^[^ ^156^ ^]^ In fact, in the last decades, there were major attempts in the development of therapies that target VEGF, such as the administration of dl‐3‐n‐Butylphthalide (NBP) in patients recovering from acute cerebral infarction, which resulted both in statistically significant increases of serum VEGF levels, as well as an enhanced recovery compared to the control group.^[^ ^157^ ^]^ Chan et al. investigated the use of VEGF165‐binding heparan sulfate sugars on rats recovering from ischaemia, and showed an increased proliferation and differentiation of NPCs, as well as improved neurological outcomes.^[^ ^158^ ^]^ Conversely, Uric Acid (UA) was tested as an angiogenesis inhibitor in a mouse model of middle cerebral artery occlusion showing reverse stroke‐related brain damage, and correlated with increased levels of VEGFA.^[^ ^159^ ^]^ The limit of these studies principally relies on administration routes, dosage, and timing. Indeed, abnormal expression or distribution of VEGF may result in the formation of angiomas — vascular tumors. Since VEGF is strictly localized in the microenvironment surrounding each producing cell, the therapeutic outcome of factor delivery, in terms of both safety and efficacy, is determined by the homogeneous expression in tissue rather than the total dose delivered. The heterogeneous expression can be the cause of aberrant angioma‐like vascular growth. To address this point, due to the high delivery capacity, gene therapy may represent an ideal method for therapeutic angiogenesis. However, it is extremely difficult to avoid heterogeneous expression levels in vivo using uncontrolled vectors, obtaining effective doses in some microenvironments, but also ineffective and toxic levels in other areas.^[^ ^160^ ^]^

VEGF‐induced angiogenesis can be modulated by pericyte recruitment through PDGF‐B. Pericytes provide signals that switch off endothelial proliferation and permeability and make the new vessels stable, that is, independent of continued VEGF stimulation. PDGFR‐B has been demonstrated to promotes peri‐infarct astrogliosis, oligodendrogenesis, and functional recovery after acute ischemic stroke in a knockout mouse model.^[^ ^161^ ^]^ Moreover, Saito et al. have shown that intravitreal injections of human recombinant PDGF‐B to zebrafish induced cell proliferation and improved their recovery after damage via needle puncture.^[^ ^162^ ^]^ Pericytes regulate endothelial function through TGF‐*β* signaling pathways. Due to its neurogenic and neuroprotective functions, TGF‐*β* has been extensively investigated for therapeutical purposes in brain injury, especially in cerebral ischemia. In 2008, Ma and colleagues demonstrated that intranasal administration of TGF‐*β*1 was able to reduce infarct volume, improved functional recovery, and enhanced neurogenesis in mice after stroke.^[^ ^163^ ^]^ Recently, Zhang et al. identified a novel potential therapeutic strategy for cerebral ischemia/reperfusion injury that relied on TGF‐B pathway. In middle cerebral artery occlusion/reperfusion rats, Alk5, known as TGF‐*β* type I receptor, was found to regulate neural plasticity and functional recovery via Gadd45b, a molecule with key role in anti‐apoptosis and DNA repair in stroke.^[^ ^164^ ^]^

The therapeutic window is fundamental when discussing TGF‐β administration. As for all the cytokines released during the neuroinflammatory response — astrogliosis, microgliosis — even the action of TGF‐*β*, can shift from beneficial to detrimental if the neuroinflammation cannot find a resolution. To this respect, Howe et al. demonstrated that delayed ALK5 inhibition can improve neurological functional outcomes, reduce gliosis and basement membrane fibrosis, and restore perivascular CSF distribution in aged mice stroke model. This could suggest a double role for TGF‐*β* signaling, where early activation may provide neuroprotection, but late activation has a detrimental effect on functional recovery.^[^ ^165^ ^]^

Aside from its neurogenic and neuroprotective functions, TGF‐*β* has been recently investigated as remyelinating factor promoting oligodendrocyte maturation in experimental autoimmune encephalomyelitis (EAE) animal model.^[^ ^166^ ^]^ The study provided new insights to understand the therapeutic mechanism by which systemic TGF‐*β*1 iss long‐standing used in the treatment of EAE and reveals the capacity of TGF‐*β*1 to contribute to neuronal networks regeneration after injury. However, the TGF‐*β* effect has been always related to the glial scar formation following CNS injuries. Conversely to PNS, this glial scar formation significantly inhibits nerve regeneration, which leads to the tissue loss of function.^[^ ^167^ ^]^ These evidences highlight the complexity of signaling molecules and should lead to a reevaluation of their functions in relation to the environment, especially for their full potential to be exploited for therapeutical applications.

Furthermore, other signaling molecules and drugs have been investigated in the context of ischemic stroke and neurovascular regeneration, and are shortly reported below.

Concerning ischemic stroke, Wnt has been proposed as a potential treatment by Menet et al.^[^ ^168^ ^]^ Wnt administration improved proliferation and migration of NPCs and enhanced CBF around the injury.^[^ ^168^ ^]^ In mice, long‐term recovery was also enhanced after delivering Wnt3a directly into their striatum.^[^ ^168^ ^]^ Intercellular Adhesion Molecules (ICAM) were studied as downstream effects of Enlimomab, an ICAM‐1 antibody found in mice. Human patients with ischemic stroke were either given Enlimomab or a placebo, and the drug resulted in worse recovery, fewer symptom‐free patients, more infections, and fever.^[^ ^169^ ^]^ Cytoflavin was tested on human adult patients, following an ischemic stroke. Those who were drug‐administered showed increased levels of BDNF and improved cognitive functions after recovery.^[^ ^170^ ^]^ Semax and aerobic exercise have also shown to increase BDNF levels in patients recovering from stroke.^[^ ^171^ ^]^ Particularly, the effects of different exercise types on BDNF levels were discussed, and it appeared that an intensity large enough to cause lactate accumulation was necessary to increase BDNF levels, suggesting a possible role for lactate in the relationship between exercise and BDNF.^[^ ^172^ ^]^ GABA receptor agonists—including Chlormethiazole or Diazepam—were shown to decrease infarct size and improve functional outcomes in animal models. However, their use is limited due to sedative side effects, and no solid proof has been found supporting their use for treatment in patients recovering from an acute stroke.^[^ ^173^ ^]^ Candesartan (ARB), an AT2‐R agonist, was tested on rats, showing decreased cognitive impairment following a stroke. The positive results were also found when treatment was delayed to 7 days post‐stroke.^[^ ^174^ ^]^ Finally, Shh has been investigated in relation to the recovery from traumatic brain injury founding that exogenous Shh reduced cerebral edema and neuronal apoptosis and promotes neural recovery in rats.^[^ ^175^ ^]^

### 5.3 Signaling Molecules and Pharmaceutical Drugs in the PNS

Less research has been conducted regarding common treatment options between vascular and neural disorders in the PNS. However, unlike CNS, injury to peripheral nerves can be recovered and functionality restored. Axon regeneration is made possible as long as the cell body is undamaged, and they have made contact with the Schwann cells in the endoneurial channel. As long as the endoneurial remains intact, it can direct axon growth back to the correct targets. However, it is essential that the axon encounters a clear path during its elongation and an unrestrictive microenvironment that promotes regeneration. Accordingly, the immediate intervention of phagocyte cells such as Schwann cells or macrophages is fundamental to clear away debris, avoiding any scar formation, and realize neurotrophic proactive factors.^[^ ^167^ ^]^ Growth factor‐based therapy is a promising strategy to succeed in nerve regeneration and functionality.^[^ ^176^ ^]^

In the neurovascular context, NGF is of particular interest because of its ability to improve both axonal regeneration and angiogenesis. Moreover, exogenous administration or NGF has been recently demonstrated to accelerate regrowth and remyelination after peripheral nerve injuries by promoting degenerative nerves collapse and myelin debris clearance.^[^ ^177^ ^]^ Slimily, NGF has been associated with PAD.^[^ ^178^ ^]^ Increased levels of NGF have been reported in limb ischemic muscles with strong similarity in terms of the time‐course with increased HIF‐1*α* levels. Interestingly, under hypoxic conditions, vascular smooth muscle cells can secrete PDGF, stimulating angiogenesis, cell migration, axonal outgrowth, regeneration, and peripheral target innervation via activation of c‐JNK and Akt phosphorylation that are required for NGF‐responsive genes activation.^[^ ^179^, ^180^ ^]^

Administration of angiogenic factors for PAD treatment has been investigated and was usually more successful when performed in combination with stem cell treatment. Stem cells can be modified to express molecular factors—including VEGF, PDGF‐ββ, FGF, and TGF‐*β*—for therapeutical engraft in vivo.^[^ ^181^ ^]^ For example, VEGF‐expressing MSCs were administered to mice suffering from CLI, resulting in a significant increase in blood flow restoration.^[^ ^182^ ^]^ Such procedures have therefore been deemed safe in animal models. However, as previously described, VEGF administration efficacy relies on distribution rather than pure quantity. Homogeneous microenvironmental distribution of VEGF has been proved to effectively restore functional blood flow in limb ischemia.^[^ ^183^ ^]^

Neuronal regeneration, outgrowth, and functional recovery are long‐lasting procedures that can takes days or months, depending on the extension of injury. Therefore, therapeutical strategies based on growth factors should take into account long treatments and efficient ways of administration. Controlled delivery systems, such as infusion pumps, biomaterial encapsulation, or multiphase loading methods, are currently used for sequential and spatiotemporal drug release, leading to the homogeneous retention of the growth factor by the region of interest with a desirable concentration.^[^ ^156^, ^184^ ^]^

The administration of growth factors coupled with technological advances has led to significant improvements to the regenerative medicine of PNS. However, much research still needs to exploit the full regenerative potential of PNS achieving still more accurate and reliable therapeutic strategies.

### 5.4 Stem Cell Therapy in the CNS

As previously mentioned, stem cells not only contribute to regenerative processes by developing into differentiated cells, but are also capable of secreting molecular mediators, and might activate yet unidentified cytoprotective and regenerative pathways.^[^ ^185^ ^]^

Multiple extensive reviews on stem cells and their use for therapeutical purposes have been proposed in a variety of neurological disorders including traumatic brain injury, and NDs.^[^ ^186^, ^187^, ^188^ ^]^ Regarding neurovascular disorders of the CNS, adult tissue‐derived stem cells, including those harvested from bone marrow, adipose, and umbilical cord, have been widely used in cell therapy, the first being usually preferred because of the long track record of their solid safety profile.^[^ ^189^ ^]^ Transplantation with bone marrow‐derived cell populations, primarily MSCs and Mono Nuclear Cells (MNCs), have been exploited in clinical trials for stroke treatment.^[^ ^190^, ^191^ ^]^ However, conflicting data emerged comparing trials leaving open interpretation on the utility of this approach. Moreover, no clear evidence has been reported regarding the molecular mechanisms behind the effective or ineffective role of stem cell transplantation. A major contributor to this discordance is the translational discrepancy between the laboratory and clinical stem cell transplant protocols.^[^ ^189^ ^]^ Strict adherence to the preclinical outcomes of optimal cell dose, timing, and administration route is essential to reach successful clinical results, as well as the availability and homogeneity of donor cells. In this respect, Adipose Tissue‐Derived Mesenchymal Stem Cells (ADMSCs) can be more easily isolated and obtained in larger amounts compared with bone marrow cells. Intravenous ADMSCs administration in rats suffering from ischaemia has shown positive results in behavioral and motor recovery, but infarct size did not differ from the control group.^[^ ^192^ ^]^ Contrary to iPSCs or embryonic stem cells, MSCs and ADMSCs are not associated with a risk of teratoma formation, because they are adult stem cells with restricted potential of differentiation. On the other hand, the pluripotency of these cells still represents a limit to therapeutical applications. Indeed, undesired cell type differentiation can elicit a deleterious immune response or generate harmful inflammatory reactions. To overcome this, multipotent stem cells such as NSCs are under investigation. NSCs are the adult stem cells of the CNS and, despite their capability of self‐renewal, their cell fate is restricted to neurons or glial cells. NSCs contribute to plasticity and brain repair, especially in injury conditions, such as trauma or ischemia.^[^ ^193^ ^]^ Because of their neurorecovery activity and safety, NSCs have been successfully applied to the therapy for stroke.^[^ ^194^, ^195^, ^196^ ^]^ Moreover, NSCs can be easily derived from iPSCs, allowing the use of donor cells of the same patient that will receive the transplantation, thus avoiding ethical issues concerning the origin of the administered human stem cells. Although promising, results achieved so far seem inconclusive, due to contradicting findings across studies and extremely limited sample sizes in clinical trials.^[^ ^197^ ^]^ A common problem when administrating cells is off‐target delivery. Administration routes and appropriate doses are fundamental to achieve enough percentage of NSCs that successfully migrate within the ischemic area. Innovative biomaterials are currently studied as vehicles to increase this percentage.^[^ ^198^ ^]^ However, to succeed the engraft donor cells need to adapt and survive under ischemic conditions. To this respect, pre‐conditioning steps such as the exposure of NSCs to an ischemia‐like environment^[^ ^199^ ^]^ or genetic modifications^[^ ^200^ ^]^ can increase cellular resistance and adaptability.

Overall, these studies underline the need to understand neurovascular signaling pathways to elucidate the molecular interplay between the two systems, but also to identify novel molecular targets or cellular treatments for therapeutical applications.

### 5.5 Stem Cell Therapy in the PNS

Administration of a variety of stem cells — including endothelial progenitor cells (EPC),^[^ ^182^, ^201^ ^]^ bone marrow‐derived mononuclear cells (BM‐MNC),^[^ ^183^, ^202^ ^]^ peripheral blood mononuclear cells (PB‐MNC),^[^ ^184^, ^203^ ^]^ or MSCs^[^ ^185^, ^204^ ^]^— has been studied in rodents and humans suffering from limb ischaemia, generally showing positive results. However, evidence found in humans is not strong enough yet, for stem cell therapy to become a standard treatment against limb ischaemia and PAD. Rigato et al., in their meta‐analysis, concluded that despite placebo‐controlled trials and randomized control trials showed no significant results, stem cell administration should be attempted in cases where no alternative is possible, other than the patients having their limbs amputated.^[^ ^205^ ^]^

A few factors have been suggested to cause the lack of strong evidence in humans. Among others, clinical studies usually targeted populations with comorbid conditions, such as diabetes, which could hinder angiogenic mechanisms even in the presence of stem cells.^[^ ^185^ ^]^ On top of that, some stem cell types, especially BM‐MNCs and PB‐MNCs, may contain a variety of subtypes, the different mechanisms and effects of which are still unclear.^[^ ^185^ ^]^

Aside from its many difficulties, NSC therapy is considered by many to be the future of regenerative strategies. According to Zhang et al., NSC transplantation was shown to improve neurological function in 36 studies, histology in 22, and both neurological function and histology in 21.^[^ ^206^ ^]^

Overall, many studies have been conducted investigating the use of stem cells as potential treatments, for a great variety of disorders. However, they often differ in the origins of the stem cells used and transplant protocols, and larger study populations are needed.^[^ ^189^ ^]^ On top of that, many of their interactions are still unclear. For example, BDNF was shown to promote the differentiation and survival of transplanted NSCs.^[^ ^206^ ^]^ Interestingly, studies have reported increased benefits of NSC therapy when combined with exercise.^[^ ^197^ ^]^ Exercise was shown to enhance contact between NPCs and the vasculature, and increase both angiogenesis and neurogenesis in the adult hippocampus. Exercise increases the concentrations of several growth factors in blood, including VEGF and BDNF, which are known mediators of neurogenesis and could therefore regulate this process.^[^ ^102^, ^207^ ^]^ Therefore, signaling molecules described across this review, acute exercise, and stem cells may interact, and studying the mechanisms through which they affect each other could be a promising focus of research for treatment against neurovascular disorder.

### 5.6 Summary

Regenerative medicine is the branch of science that develops strategies to repair and regenerate cells, tissues, or organs. The understanding of neurovascular interplay is essential, as innervation and vascularization are common fundamental traits of every tissue or organ of the human body. Two main regenerative approaches aimed at restoring neurovascular disorders are currently under investigation in the therapeutical research field.

The first exploits the direct administration of exogenous ‐ or of drugs that target endogenous ‐ growth factors or signaling molecules that can stimulate neurogenesis and angiogenesis within CNS and PNS, that is, mainly VEGF, PDGF‐ββ, TGF‐β, and NGF respectively. The limit of these studies principally relies on administration routes, dosage, distribution, and interaction with the surrounding microenvironment. Timing and way of administration are crucial as well, because of the length of repair and regeneration processes. Fortunately, thanks to technological advances, increasing and more accurate therapies are emerging. Such treatments promise bright expectations for translation therapy on humans.

The second takes advantage of the stem cell injection to physically replace damaged tissue and chemically restore the microenvironment balance through the release of neurotrophic and neuroprotective molecules. Adult tissue‐derived stem cells, including MSCs, MNCs, and ADMSCs, have been widely used in cell therapy for cerebrovascular disorders. As adult stem cells, they are not associated with a risk of teratoma formation, because of their restricted potential of differentiation. On the other hand, their pluripotency still represents a limit to therapeutical applications in terms of undesired cell type differentiation. NSCs overcame these limits as adult multipotent stem cells with a restricted and specific differentiation fate. Therapeutic attempts with adult stem cells have been declared safe and are currently under investigation. Administration routes, appropriate doses, and cell graft survival are crucial. Although promising, results achieved so far seem inconclusive due to contradicting findings across studies, and extremely limited sample sizes in clinical trials. Thus, the effort of researchers is now focusing on clearing the translational discrepancy between pre‐clinical and clinical research in order to assess the efficacy of stem cell therapy.

## 6 Conclusion

This review assessed signaling pathways in the developing vascular and neural systems, and reviewed their current uses in regenerative medicine. Comparing both systems can bring to light molecular processes of special interest in the context of neurovascular regeneration. FGF, BMP, Slit/Robo, and Shh were shown to participate in both the developing vascular and neural systems, but have not yet been studied at the neurovascular level. VEGF, SEMA/Plexins/NRP, and Ephrins were discussed in all three sections and could therefore be especially promising topics of research. Indeed, future research could improve existing therapies for neurovascular disorders by investigating molecular mechanisms of angiogenesis and neurogenesis in more depth. Observing the effects of different guidance cue combinations on tip cells and growth cones in vitro, and analyzing the mechanisms modulating the balance between development and quiescence in endothelial and neural cells, could greatly better our understanding of the mechanisms at play in recovery.

Overall, neurovascular regeneration is a rapidly growing and promising field of research. Although its mechanisms are extremely complex and involve a vast range of cell types and signaling pathways, investigating the similarities and interactions between the vascular and nervous systems would bring valuable insight and should be a focus of future research.

## Conflict of Interest

The authors declare no conflict of interest.
